# Photosynthetic protein classification using genome neighborhood-based machine learning feature

**DOI:** 10.1038/s41598-020-64053-w

**Published:** 2020-04-28

**Authors:** Apiwat Sangphukieo, Teeraphan Laomettachit, Marasri Ruengjitchatchawalya

**Affiliations:** 1 Bioinformatics and Systems Biology Program, School of Bioresources and Technology, King Mongkut’s University of Technology Thonburi (KMUTT), Bang Khun Thian, Bangkok 10150 Thailand; 2 Biotechnology program, School of Bioresources and Technology, KMUTT, Bang Khun Thian, Bangkok 10150 Thailand; 3 School of Information Technology, KMUTT, Bang Mod, Thung Khru, Bangkok, 10140 Thailand; 4 Algal Biotechnology Research Group, Pilot Plant Development and Training Institute (PDTI), KMUTT, Bang Khun Thian, Bangkok, 10150 Thailand

**Keywords:** Computational biology and bioinformatics, Gene ontology

## Abstract

Identification of novel photosynthetic proteins is important for understanding and improving photosynthetic efficiency. Synergistically, genome neighborhood can provide additional useful information to identify photosynthetic proteins. We, therefore, expected that applying a computational approach, particularly machine learning (ML) with the genome neighborhood-based feature should facilitate the photosynthetic function assignment. Our results revealed a functional relationship between photosynthetic genes and their conserved neighboring genes observed by ‘Phylo score’, indicating their functions could be inferred from the genome neighborhood profile. Therefore, we created a new method for extracting patterns based on the genome neighborhood network (GNN) and applied them for the photosynthetic protein classification using ML algorithms. Random forest (RF) classifier using genome neighborhood-based features achieved the highest accuracy up to 87% in the classification of photosynthetic proteins and also showed better performance (Mathew’s correlation coefficient = 0.718) than other available tools including the sequence similarity search (0.447) and ML-based method (0.361). Furthermore, we demonstrated the ability of our model to identify novel photosynthetic proteins compared to the other methods. Our classifier is available at http://bicep2.kmutt.ac.th/photomod_standalone, https://bit.ly/2S0I2Ox and DockerHub: https://hub.docker.com/r/asangphukieo/photomod.

## Introduction

Photosynthesis is a multistep process comprising solar energy harvest, excitation energy transfer, energy conversion, electron transport, in particular, the transport of electrons flow from water to NADP+ and other electrochemical energy via photosystems and a series of enzymatic reactions required to synthesize components for cellular metabolism and environmental adaptation. Recently, photosynthetic prokaryotes, in comparison with plants, have attracted considerable attention for a wide range of renewable energy, agricultural and environmental applications aiming for sustainable development^1^, probably due to their excellent productivity. However, it has been reported that the photosynthetic conversion efficiency falls around 6% of the total incident light. Maximizing photosynthetic efficiency is a major challenge in the current efforts to manage and/or engineer photosynthetic organisms^2^. To achieve this goal, all photosynthetic components and their roles in photosynthesis need to be clarified beforehand.

Basically, photosynthesis-related genes can be identified by two mains approaches: experimental and computational approaches. Although experimental approaches have successfully been used to identify many components^3^, they can be expensive and time-consuming. Besides, many photosynthetic components are temporarily present^4^, and their deletion might have no effect on photosynthetic growth^3^. These limitations push forward the development of various computational approaches. These approaches have been used to narrow down and specify gene function before the validation by expensive experimental approaches. Sequence similarity search is conventionally used for gene annotation, and its validity depends on prior knowledge. By searching against known protein databases, (i.e. NCBI databases), proteins involved in photosynthesis are hypothetically annotated with unknown functions and many times misidentified^5^. It has been shown that 70% of close homologs of photosynthetic proteins come from non-photosynthetic organisms^6^, resulting in unsatisfactory results in attempt to identify photosynthetic proteins. Unlike the conventional approach, ML provides insight into the functional classification of novel proteins with no homology to proteins of known function^7^. Various ML methods have been developed with different learning features and algorithms. SCMPSP^8^ is a scoring card-based method, which calculates the propensity scores of amino acids and dipeptides from a set of photosynthetic and non-photosynthetic proteins and generates the model using a genetic algorithm. The model also reveals general characteristics of photosynthetic proteins, such as the preferable presence of amino acids containing hydrophobic side chains, amino acids prone to forming helices in membranes, and exhibiting low interaction with water. SVMprot^9^ uses a support vector machine (SVM) to learn physicochemical features, such as hydrophobicity, polarity, charge and solvent accessibility, for each amino acid residue of representative proteins to predict protein functions including photosynthetic functions. It was applied to discover several novel photosynthetic proteins in plants^10^. Recently, DeepGO^11^, a large-scale protein function prediction method, has been used. It employs the recent advanced algorithm, deep neuron networks in combination with protein-protein interaction networks to predict protein functions including photosynthetic function. However, poor prediction performance, especially in relation to the biological process^12^, has necessitated the use of additional information such as gene cluster to infer protein functions^13^.

Analysis of prokaryote genomes has revealed that genes, including those involved in the photosynthesis, tend to form clusters^5,14^. It has been suggested that genes located in the same cluster participate in the same biochemical network^15^ or exhibit the same expression pattern^16^. Thus, the analysis of such conserved clusters provides useful information for gene annotation and genome evolution. Several methods have been developed to uncover conserved gene clusters and identify the functional connections in such a group of genes^14,17,18^. The general approach starts by identifying an orthologous set of a given gene in all available genomes. Next, the neighboring genes are extracted and ranked by the frequency of their occurrence. The neighboring genes are expected to have a functional connection to the orthologs if there is a statistically significant association between them^19^. Instead of using the number of occurrences, a tree-based probabilistic method^20^ that shows better performance has been developed. However, the difficulty in handling large scale data limited its application. Lately, GNN was shown to cope with large scale prediction of enzymatic activities of uncharacterized enzymes^18^. However, the use of this method requires prior knowledge of the protein families and complicated interpretation^21^.

Therefore, we applied the ML approach integrated with genome neighborhood-based features to facilitate the photosynthetic function assignment. In the first section of this study, we observed functional relationships between photosynthetic genes and their neighbors to determine the possibility of using information from neighboring genes to infer the photosynthetic function. Next, gene neighborhood patterns were extracted by our newly developed method based on GNN. The patterns were applied as features for learning by ML algorithms. Then, the new model, named as ‘PhotoMod’ (Photosynthetic genes/proteins classification Model), was fine-tuned until it reached the highest performance. The application of the model was demonstrated by comparing the prediction performance with other available tools and also, by predicting 12 novel photosynthetic proteins, which were recently identified by experiments^22–28^.

## Material and Methods

### Collection of photosynthetic proteins using photosynthesis-specific GO terms

Supplementary Tables that contain at least one of 61 photosynthesis-specific GO terms reported by Ashkenazi *et al*.^6^ (Supplementary Table S1) were collected from the UniprotKB/Swiss-Prot database (until September 2016) as an initial positive dataset. In total, 15,195 unique proteins labeled with at least one of 61 GO terms were found, confirming their photosynthetic function. The photosynthetic function of the initial positive dataset was transferred to our protein dataset, which is a complete set of proteomes of 154 photosynthetic prokaryote genomes (Supplementary Text S1). The photosynthetic function was transferred if a percent identity and percent sequence coverage of more than 80% were obtained. Finally, the positive data set contained 6,430 protein sequences. The negative dataset was collected from non-photosynthetic genes in UniprotKB/Swiss-Prot. The protein sequences from UniprotKB/Swiss-Prot that were not labeled with any photosynthetic GO term were randomly selected. To ensure the absence of photosynthetic function in the negative dataset, all ancestor nodes of annotated GO terms in each sequence were retrieved from the GO.db library. Sequences containing GO nodes that matched the photosynthetic function were removed. Additionally, the sequences were necessarily annotated at least to GO level 3, which is the same level of photosynthesis (GO:0015979); otherwise, they were automatically removed. The sequences that passed the criteria were blasted against our protein dataset from 154 genomes with stringent criteria (percent identity and coverage >80%)^29^. The non-photosynthetic dataset was randomly selected to be equal to the number of positive dataset from the pool of matched protein sequences. The datasets of both photosynthetic and non-photosynthetic proteins were clustered to reduce sequence redundancy to lower than 25% identity.

### Gene neighborhood calling

**Figure 1:**
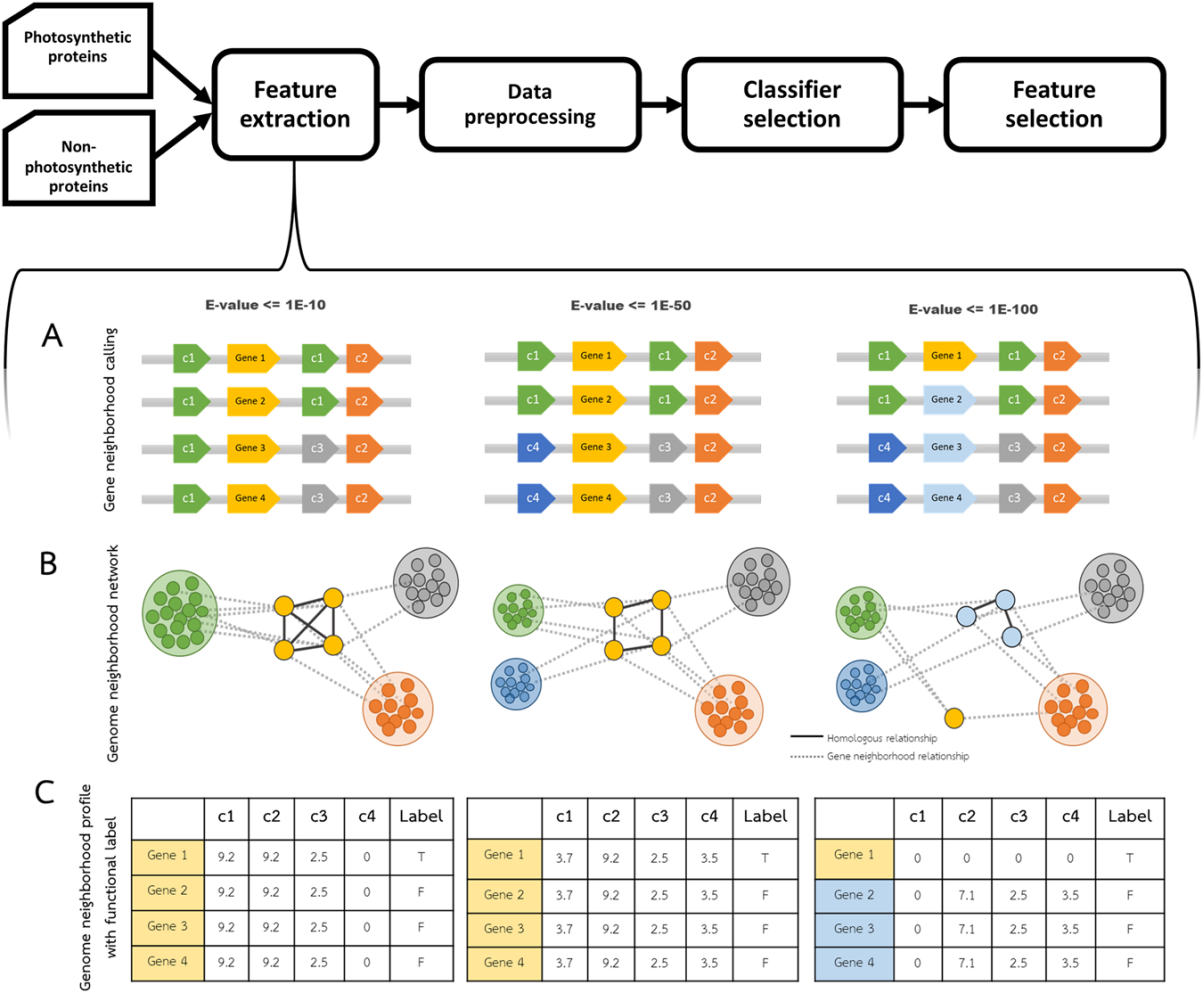
Protocol for building the model of photosynthetic protein classification and the demonstration of the feature extraction method. The protocol consists of dataset building, feature extraction, data preprocessing, classifier selection and feature selection. (**A**) Genome neighborhoods are called by using intergenic distance criteria in the feature extraction step. Genes in the same homologous group are indicated by the same color label. The query genes conserved in different four genomes are labeled by Genes 1-4. (**B**) Relationships between query genes and their neighbors are displayed in GNN. Straight-line represents a homologous relationship, while the dashed line represents genome neighborhood relationships. Color labeled on each node represents a homologous group corresponding to gene color in (**A**). (**C**) The genome neighborhood profile of each query gene is represented in table format. The value in the table is the Phylo score, which represents the level of gene neighborhood conservation. E-values (1E-10, 1E-50 and 1E-100) are the thresholds for family classification of proteins coding from the genes.

As described in Supplementary Text S1, we retrieved 154 completed photosynthetic prokaryote genomes from the NCBI database. Genes in each genome were identified and an in-house python script was used for the selection of neighboring genes in each genome. Genes on the same strand were considered neighbors if they were within an intergenic distance of not more than 250 bp^30^ or overlapped each other (Fig. 1A). In addition, when the gap between the first genes of two neighborhood gene clusters with divergent directions is within 200 to 1000 bp—regarding the operon interaction concept shown in *Escherichia coli*^31^—, the two clusters were merged into the same neighborhood gene cluster.

### Genome neighborhood network

Sequence similarity network (SSN) was applied to discriminate the protein function^32^ using different stringent E-value thresholds to construct the group-wise sequence similarity relationships. This approach enables functional and evolutional analysis of large protein datasets and is also applied to genome neighborhoods to guide the discovery of novel metabolic pathways^18^. In this work, three different Blastp E-value cutoffs (1E-10, 1E-50 and 1E-100) were employed for all proteins from the 154 genomes. Then the Blastp outputs were used as inputs for the Markov cluster algorithm (MCL) to cluster proteins into families (Supplementary Text S1). Figure 1B visualizes the genome neighborhood networks, where homologous relationships from the SSN at different E-value cutoffs were integrated with gene neighborhood relationships from the gene neighborhood calling step.

### Genome neighborhood profile generation

The genome neighborhood networks are transformed to the table form called genome neighborhood profile (Fig. 1C), which consists of i) query proteins in the first column, ii) the genome neighborhoods cluster ID columns containing the value of ‘Phylo score’ and iii) the functional label in the last column. Due to the imbalance in the phylogenetic diversity of the genomes in the dataset, we developed the ‘Phylo score’, modified from the conservation scores C_u_ and C_d_ from Zheng’s method^20^, which incorporates the information captured by a genome phylogenetic tree to overcome the bias of dominant organisms in the detection of conserved gene clusters. We use this method to determine the level of genome neighborhood conservation. The Phylo scores of neighboring genes were calculated if they are conserved with the query genes in at least three genomes. The calculation of Phylo scores (Supplementary Fig. S1) involved three main steps: (i) performing bidirectional Blast best hit of all pairwise genomes, (ii) generating a pairwise matrix of a proportion of shared gene content, and (iii) constructing a phylogenetic tree using genomes containing conserved gene neighbors. Details is described as the follows.

Pairwise comparisons of all 154 proteomes (Supplementary Table S2) were performed using Blastp and the shared gene content of each pair of genomes was identified by reciprocal best hit, implemented by an in-house python script. The pairwise distance between genomes was calculated by *d* = *-ln(s)*, where *s* is the proportion of shared gene content of the two genomes divided by the average number of proteins between the two genomes. Then, the phylogenetic tree of the genomes, in which the gene neighbors were identified, was constructed using the pairwise distance profiles that were created, i.e. genome distance matrix. Biopython package in Python (Bio.Phylo.TreeConstruction) was used to generate the distance matrix and the phylogenetic tree. The phylogenetic tree was built by the neighbor-joining algorithm. The Phylo score was determined by the summation of the total branch length of the tree.

When the stringency was increased from 1E-10 to 1E-50, the neighboring genes of the query genes (Genes 1-4) split from three to four clusters as shown by GNN (Fig. 1B). As a result, no Phylo score was assigned to c1 because the Phylo score was calculated (neighboring genes are considered conserved) only if genes are found as neighbors in at least three genomes. In addition, a new cluster (c4) that was split from c1 was added with a corresponding Phylo score. When the E-value was increased to 1E-100, the cluster of the query separated into two clusters (yellow and light blue clusters). Gene 1 became the only member of the yellow cluster and as a result, no Phylo score was assigned. Accordingly, the Phylo score of the gene cluster c2 of the light blue query decreased because the number of genomes that the light blue cluster genes and c2 cluster genes are conserved decreased from four to three.

The last column in the table is the label of the protein function (T = photosynthesis, F = non-photosynthesis). As demonstrated, the low stringent E-value yields similar patterns of neighboring genes; thus, it was impossible to separate the protein functions. A significant difference in the patterns was observed in more stringent criteria. Thus, it was expected that when the three tables are combined together, they can separate the function of the query proteins. The point of this example is to show how patterns of neighboring genes separate the true gene function from the background noise, even when the query sequences are closely related.

Practically, the Phylo score is numeric and diverse depending on the distance of the genomes; thus, to make it easier to be understood by the machine, normalization was applied. Quartile was carried out to discretize all Phylo scores (from the entire neighboring gene of photosynthetic genes) to four levels. Phylo scores in Level 0 (Phylo score = 0), level 1 (first quartile), level 2 (second quartile) and level 3 (third quartile) indicate no gene conservation, low gene conservation, intermediate gene conservation, and strong gene conservation, respectively. After the process was computed, the Quartile 1 and Quartile 3 cutoff points of 0.41079 and 2.61799 respectively were found. The normalized Phylo score tables from different E-value criteria were combined and converted into ARFF format (via Weka platform).

### Construction of PhotoMod, a GNN-based model

*Photosynthetic and non-photosynthetic protein dataset building*. This first step involves generating input data as described above.

#### GNN feature extraction and data preprocessing

This second step involves (1) calling gene neighbors, (2) measuring Phylo score and 3) normalizing the Phylo score (see part Gene neighborhood profile generation). Data preprocessing was carried out by removing redundant data resulting in 1,029 unique instances (257 photosynthetic instances and 772 non-photosynthetic instances) from the total 12,858 instances. A total of 18,849 features, cluster IDs, were obtained. This dataset represents two common problems encountered in real-world classification namely class imbalance and high-dimensional data. We carefully considered these problems by choosing the evaluation metric (Supplementary Text S2) and classifiers that could attenuate these problems.

#### Classifier selection

Three different classifiers were included in this third step according to their prediction performance on high-dimensional data^33,34^. The first was a probabilistic-based classifier called Bayesian Network (BN)^35,36^, which has a structure in the form of a directed acyclic graph and allows attribute-attribute interactions, thereby improving classification performance, especially for large datasets. The second was a support vector machine (SVM)-based classifier named Sequential Minimal Optimization (SMO), which is known to handle high-dimensional data, such as the microarray classification, well^37^. The last algorithm was a decision tree-based classifier named random forest (RF)^38^, which uses the vote from multiple decision trees. The best performing classifier that is most suitable to our data type was determined by the 10-fold cross-validation (CV) method.

#### Feature selection

The last step of the model development involved model optimization, by which the optimal feature subsets were determined to reduce computational complexity and the chance of fitting noise in the learning algorithm. Two different schemas were applied to the selected best-performing classifier. Firstly, the features were initially filtered by the Gain Ratio (GainRatio) approach, which is able to evaluate how each feature contributes to decreasing the overall entropy in the dataset. Secondly, principal component analysis (PCA) was used to reduce the dimensionality of the dataset, while still retaining most of the information.

### Blastp and SCMPSP models constructions

In the case of Blastp, the test dataset was used as a query to blast against the database that was built by the training dataset. The best result was used to compare after varying E-value parameter. The SCMPSP model was set up according to Vasylenko’s method^8^ and the best model after the optimization process was used to compare (Supplementary Fig. S2).

## Results

### The functional relation between photosynthetic genes and their neighbors

The functional relationship between photosynthetic genes and their neighbors was analyzed in order to determine the possibility of assigning a photosynthetic function based on the neighboring gene profile. Curated gene ontology (GO) profile of photosynthetic genes of seven reference genomes (Supplementary Table S3) retrieved from Uniprot was used as the curated dataset. We then investigated the relationship between the Phylo scores−a conservation score of neighboring genes−and a higher prediction specificity achieved when a more stringent criterion is used, and their functional similarity, in terms of GO. The F1 measure, which is a harmonic average between precision and recall, is used as a function-related evaluation method, and it can evaluate how much overlap there is between the GO labels of the photosynthetic queries and their neighbors. High F1 measure indicates high similarity between the GO term of a query and that of its neighbors, while low F1 measure indicates low similarity. To avoid incomplete annotation, all ancestor nodes of labelled GO terms were retrieved via GO.db, and redundant GO terms were removed.

**Figure 2:**
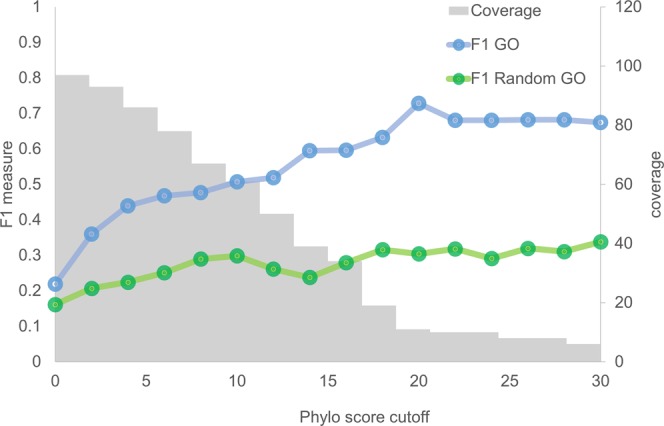
The relationship between conservation score (Phylo score) (x-axis) and F1 measure (y-axis) of *Prochlorococcus marinus*. The blue line represents the F1 measure for indicating the similarity between GO terms from photosynthetic genes and from their neighbors. The green line represents the F1 measure for indicating the similarity between GO terms from photosynthetic genes and from random GO terms. Coverage (right x-axis) represents the number of predicted proteins.

The results of the GO profile analysis of the seven genomes were similar, with respect to trend (Supplementary Fig. S3). Figure 2 shows the results derived from the *Prochlorococcus marinus* genome, as an example. We generated a random GO dataset from the pool of all GO terms annotated from all proteins in the reference genomes. The random dataset was sampled with a number equal to the number of GO terms in each conserved neighbor criteria. Without a Phylo score cutoff, the similarity between the GO terms of photosynthetic queries and their neighbors was low and almost equal to the random GO terms, probably due to the inclusion of neighboring genes that are not related to the photosynthetic function in the calculation. We found that when the Phylo score cutoff was increased, the F1 measure accordingly increased and was higher than that of the random GO dataset. This result indicates that the functional relationship is not due to chance. This evidence suggests that gene neighbor conservation is a feature that can be used for functional assignment. However, we found that prediction coverage is a limitation of this method. As shown in the graph, the prediction coverage dropped continuously when the cutoff became more stringent. Therefore, it became clear that this method needed more systematic ways to guide functional assignments.

### GNN-based machine learning model development for photosynthetic protein classification

As the Phylo scores represent conserved gene neighborhoods with potential functional similarity, we proposed that by using the conservation profile of gene neighborhoods, photosynthetic genes/proteins can be distinguished from non-photosynthetic genes/proteins. Considering the numerous benefits of employing ML to predict protein function, the algorithms were performed using Weka software version 3.9.1^39^. The protocol for building the model of photosynthetic gene classification namely PhotoMod (PHOTOsynthetic MODel), as shown in Fig. 1, contains information on dataset building, feature extraction, data preprocessing, classifier selection and feature selection.

The performance of three different classifiers, BN, SMO and RF was evaluated and compared after fine-tuning parameters (Supplementary Table S4). It was found that RF showed the highest performance with 88% accuracy (Table 1). Also, the F1 measure of minor class and MCC indicated that RF did not suffer from the dataset imbalance as much as the other methods, especially, BN. SMO performed well and better than BN, which was the worst classifier in all aspects. However, the average overall performance of the three classifiers, >80% of accuracy, indicates that the gene neighborhood profile can be used to discriminate between the photosynthetic function and non-photosynthetic function.

**Table 1:** Classification performance of different classifier algorithms and class balancing methods.

Class balancing methods	Classifiers	Accuracy	F1 minor	MCC
No filter	BayesNet	0.829	0.562	0.533
	RandomForest	0.883	0.778	0.702
	SMO	0.872	0.771	0.685
SMOTE	BayesNet	0.845	0.614	0.581
	RandomForest	0.880	0.778	0.698
	SMO	0.857	0.753	0.658
ClassBalancer	BayesNet	0.833	0.571	0.546
	RandomForest	0.858	0.758	0.664
	SMO	0.853	0.753	0.656

To address the class imbalance problem that was found during the data preprocessing step, two supervised approaches were introduced. First, the ClassBalancer method was applied to reweight the instances of each class in the dataset equally, while the total sum of weights of all instances was maintained. The second method involved the resampling of the instances of the minor class in the dataset by applying the Synthetic Minority Oversampling TEchnique (SMOTE)^40^. It was found that the two approaches were able to improve the prediction performance of BN, but not SMO and RF (Table 1). The prediction performance of BN was improved in all aspects when ClassBalancer and SMOTE were applied. Both filter methods decreased the classification performance of RF classifier, which comes as no surprise because tree-based algorithms often perform well on imbalanced datasets^41,42^. Also, it has been previously shown that many classifiers, including RF, do not benefit from SMOTE when high-dimensional datasets are used^43^, but it is not known why supervised balancing approaches struggle with high-dimensional class-imbalanced data^43^. Our result suggests that RF is the most suitable approach to deal with high-dimensional class-imbalanced data without any boosting method.

In order to make the model more robust and reduce the chance of overfitting, GainRatio and PCA were applied to select the optimal feature subset and reduce feature dimension, respectively, in the RF model. The result shows that the prediction performance of RF was slightly improved when the feature selection methods were applied (Supplementary Fig. S4). The improvement was observed when the number of selected features was at least 2,000. Decreasing the number of features below 100 lowers performance, which might result in the loss of important information. The features that were selected for the model tree were considered important for photosynthesis. Although three E-value criteria were applied for the protein clustering features, the most informative criterion was 1E-10 as indicated by the number of selected features (58%) as shown in Table 2. However, to achieve the highest performance, the three E-value criteria were combined. The importance of such combination was emphasized by rebuilding the model using features from a single E-value criterion and their different combinations and comparing the F1 measures of the models (Supplementary Table S5). The maximum performance was observed when all three E-value criteria were combined, which confirms our hypothesis that the three E-value criteria support each other, thereby enabling a better photosynthesis function classification. Although the E-value criterion of 1E-10 mainly contributed to the prediction performance, the lower E-values (1E-50 and 1E-100) are likely necessary for deeper function classification. After PCA was applied with 95% of the variance, the total number of features dropped to 301 features. The model performance was observed and compared to other methods as shown in the next section.

**Table 2:** The number of selected features for each E-value criterion.

E-value criteria	Number of selected features
1E-10	1,156 (58%)
1E-50	643 (32%)
1E-100	201 (10%)
Total	2,000

### Performance comparison of photosynthetic function classification methods

The prediction performance of our genome neighborhood-based model (PhotoMod) was compared with those of the basic sequence similarity approach, Blastp, and the advanced machine learning approach, SCMPSP (Supplementary Fig. S2). The performance of all three methods was evaluated using two nested 10-folds CV, where the data is split into 10 outer folds and then two inner folds are created in each outer training set to find the best parameter set to train the model before validating with the outer test set. The inner nested fold CV prevents the leak of information during the model optimization process and the bias of model comparisons. The accuracy score indicates overall correct prediction, while F1 minor is used to describe the correctness of the minor class (photosynthetic class). MCC is used to evaluate the overall model correctness in regard to its robustness to imbalanced datasets. Blast methods achieved accuracy, F1 minor and MCC up to 0.818, 0.499 and 0.447, while SCMPSP achieved up to 0.768, 0.506 and 0.361, respectively (Table 3). The two evaluation metrics, accuracy and MCC, indicated that Blast method performed better than SCMPSP significantly (P < 0.05), while F1 minor showed no significant difference (Supplementary Table S6). PhotoMod outperformed both methods significantly (P < 0.01) and achieved accuracy, F1 minor and MCC of 0.874, 0.736 and 0.718, respectively. Also, the high score of F1 minor and MCC scores indicates that our model handles imbalanced datasets well.

**Table 3:** Performance comparison among different methods in the classification of photosynthetic proteins using two nested 10-fold cross-validation.

Prediction methods	Accuracy	F1 minor	**MCC**
Blastp (sequence similarity search)	0.818 ± 0.032	0.499 ± 0.080	0.447 ± 0.090
SCMPSP (sequence-based model)	0.768 ± 0.033	0.506 ± 0.060	0.361 ±0.078
PhotoMod (genome neighborhood-based model)	0.874 ± 0.016*	0.736 ± 0.043*	0.718 ± 0.042*

*Significant difference (P < 0.01) compared to other methods tested by Wilcoxon signed-rank tests

### Performance comparison of photosynthetic function classification methods using novel photosynthetic protein dataset

The 12 novel photosynthetic proteins were collected from the literature. These photosynthetic proteins were recently reported and had never been deposited in any database before September 2016 regarding our dataset retrieval (Supplementary Table S7). To measure false positive error, we also included non-photosynthetic proteins. The non-photosynthetic dataset was collected from the recently reported prokaryotic protein sequences that contain no photosynthetic GO term label (as described in the method section), which were obtained from UniprotKB (in 2018). The protein sequences were clustered to reduce sequence redundancy to lower than 25%. In total, only 111 non-photosynthetic protein sequences passed this criterion.

The performance of the present model was compared to the other functional prediction tools that rely on amino sequence property. To enable a fair comparison, protein sequences were used as input for all prediction tools. In our case, the sequences were blasted against the protein database retrieved from the 154 proteomes. The matched sequences were used to call genome neighborhood profiles and make a prediction. A prediction class was assigned by the majority vote. The prediction performance of our model was compared with that of other prediction tools: Blastp, SCMPSP, DeepGO, and SVMprot under optimal conditions (PhotoMod E-value < = 1, Blastp E-value < = 10 and SVMProt probability > = 50%) (Supplementary Table S8).

The result in Table 4 shows that only three of 12 novel photosynthetic proteins could be assigned photosynthetic function by Blastp (see full prediction result in Supplementary Table S9) with F1 minor (0.188) and MCC (0.078) indicating that the overall performance of this method was inefficient in predicting the photosynthesis class. However, this method resulted in a high accuracy score (0.789) and a low number of false positives. On the other hand, SVMprot predicted six proteins correctly as photosynthetic proteins and allowed a higher number of false-positives than Blastp. Though, the precision of this method (0.140) was lower than the Blastp approach, but the overall performance was better (MCC = 0.104). We found that DeepGO could not be compared, because no GO term related to photosynthesis was observed. SCMPSP performed worst compared to the other methods (MCC = −0.042). Only three of 12 photosynthetic proteins were correctly predicted, while the F1 minor was only 0.120. In this comparison, PhotoMod showed a better prediction performance in terms of both F1 minor (0.300) and MCC score (0.214). Out of five prediction methods, PhotoMod and SVMprot yielded the highest number of true positives (6 out of the 12 photosynthetic proteins). However, our PhotoMod performed better on the prediction of true negatives (89 predicted correctly out of 111) compared to that of SVMprot (74 predicted correctly out of 111). Therefore, in comparison to the other methods, PhotoMod is the most applicable tool for the real situation where ones seek for a small number of photosynthetic proteins from a pool of non-photosynthetic proteins.

**Table 4:** Performance comparison of different methods in the classification of novel photosynthetic proteins.

Methods	TP	TN	FP	FN	Precision	Recall	Accuracy	F1 minor	MCC
Blastp	3	94	17	9	0.150	0.250	0.789	0.188	0.078
SVMProt	6	74	37	6	0.140	0.500	0.650	0.218	0.104
DeepGO	0	111	0	12	—	—	—	—	—
SCMPSP	3	76	35	9	0.079	0.250	0.642	0.120	-0.042
PhotoMod	6	89	22	6	0.214	0.500	0.772	0.300	0.214

**Figure 3:**
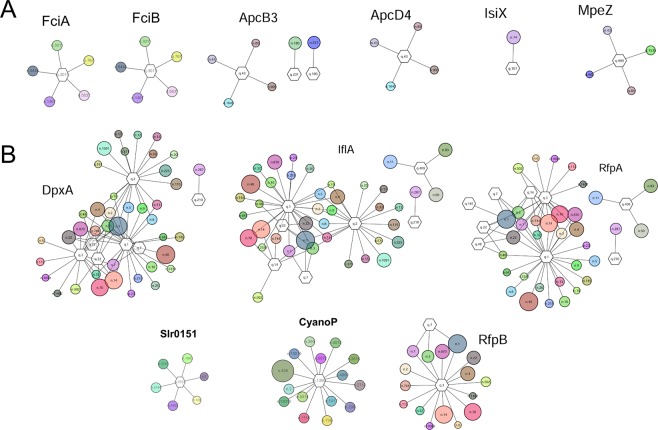
Genome neighborhood networks of novel photosynthetic genes used in this study. The protein clusters that are matched by the query sequence are represented by a hexagon. The circles represent protein cluster from the neighboring genes of which edges show gene neighborhood relationships with the query cluster. The size of the circle indicates the conservation (corresponding to Phylo score). The proteins are clustered with E-value cutoff 1E-10. The prediction results of these 12 proteins are shown in Supplementary Table S8.

The prediction results of 12 novel photosynthetic proteins from PhotoMod were further investigated to identify the sources of prediction errors by using GNN visualization. We found that the networks of 12 proteins could be seemingly divided into two groups and related to the prediction errors. The first group (Fig. 3A) contains the proteins that were classified as true positive by PhotoMod, whereas the second group (Fig. 3B) contains the proteins that were classified as a false negative. The number of conserved gene neighborhoods in each protein of the first group was obviously less than that of the proteins in the second group. The high variety and the vast number of the gene neighborhood nodes in the second group indicate that the query proteins may contribute to several pathways, and might be the reason of the false negatives. Besides, those false negative queries may contain (1) multiple domains, which can either contribute to one independent function or interact with other domains to do other functions^44^, or (2) a broad function domain such as kinase domain. It has been found that RfpA has a histidine kinase domain, which acts as sensor kinase in the signal transduction cascade^45^. DpxA contains one ubiquitous GAF domain and one histidine kinase domain for light color sensing^46^. IflA consists of three GAF domains that act as photoreceptor^23^. These domains are commonly found in the non-photosynthetic function, as predicted by sequence-similarity based methods, and likely contributed to the high variation in the GNN.

### Prediction of unknown proteins in cyanobacteria genome

More than 50% of protein-coding genes in the genome of the model photosynthetic organism, *Synechocystis* sp. PCC 6803, are unknown. We applied our platform to identify photosynthetic related genes in this organism, using the unknown protein sequences as input. Of the 1,885 unknown protein-coding genes, 479 sequences (~26%) were found to be involved in photosynthesis (Supplementary Table S10) and were validated using the information obtained from the literature. For example, the genes, *slr0144, slr0145, slr0146, slr0147* and *slr0149*, which were predicted to be related to photosynthesis by PhotoMod, have been shown to be involved in the assembly of Photosystem II in cyanobacterium *Synechocystis* sp. PCC 6803^3^. This gene set is visualized as genome neighborhood network in Supplementary Fig. S5. Moreover, it was observed that these genes are conserved, and the expression patterns are correlated with those in various photosynthetic prokaryotes^47^.

## Discussion

We showed that using phylogenetic distance criteria to assign protein function from gene neighborhood yields a high-sensitivity result, though the precision was very low. This particular event was also observed in the relaxed condition of gene cluster detection for functional classification^48^. It has been also shown that ML efficiently assigns protein function^49^ and predicts gene clusters that holds functional coupling^50^. In this study, we thus applied the ML approach with gene neighborhood-based features to facilitate the photosynthetic function assignment. The ML algorithms worked well with our feature extraction scheme, and the high classification performance of all classifiers indicates the feature merit.

High-dimensional data was observed after extracting genome neighborhood information. The RF algorithm showed the highest performance, although SVM and BN have been more popular for high-dimensional data such as microarray classification^51^ and text classification^52^. Interaction between attributes and the combination of tree predictors might be the key advantages of RF over other algorithms. It has been shown that RF performs well in the studies of interactions between genetic loci^53,54^. Although RF classifier handles high-dimensional data well, redundant and useless attributes can cause time wastage for both training and testing processes. In this study, GainRatio, a feature selection method, was employed, although it ignores feature dependencies and interaction with the classifier. Nevertheless, it benefits from improved computational efficiency, enabling high-dimensional dataset analysis^55^.

We demonstrated that our model outperforms the sequence-based methods. Basically, the sequence-based model benefits from amino acid properties that are conserved among the group of protein. However, the photosynthesis is a complex system consisting of many different proteins; therefore, broad sequence property can cause low precision. Only three of 12 novel proteins could be correctly assigned photosynthetic function by Blastp, which comes as no surprise considering the difficulty in assigning photosynthetic function by sequence-similarity search^6^. We found that DeepGO could not predict any GO term that is related to photosynthesis, suggesting that DeepGO is not efficient for specific functions, e.g. photosynthesis, because it has been trained by a large number of GO terms. Also, it is reportedly not suited for predicting specific protein functions in a biological process^11^. SCMPSP was shown previously to perform better than Blast method and be comparable to the other ML approaches^8^. However, in this study, we found that SCMPSP performed inefficiently and even worse than the Blast method. We suspect that the performance of this method depends on the genetic algorithm which might get stuck in local minima if the number of generations is not sufficiently long enough. While sequence-based models yield a low precision score, our PhotoMod combines sequence property and GNN as learning features to achieve a higher precision result. This result might indicate that the photosynthetic function is governed by genomic constraints in addition to amino acid property. Nevertheless, the false-negatives of this method result from the diverse patterns of the GNN profiles. This problem was also observed in the classification of heterogeneous data on the complex disease^56,57^. Probably, reducing the number of patterns using the clustering algorithm before ML step^57^ might be able to solve the problem.

Nevertheless, limitations of our approach should also be mentioned. First, it works well with only prokaryotic proteins, as the proteins were extracted from only photosynthetic prokaryotes. Photosynthetic eukaryotes such as plants were excluded as the concept of gene neighborhood in eukaryotes is complicated due to their chromosome conformation^58,59^. Another limitation is that if the query gene is unique, it is impossible to find conserved neighboring genes. When the queries did not match sequences in our database, which contains all proteins in photosynthetic organisms, we assumed that the sequences are not from a photosynthetic organism and discarded them to avoid misclassification. Lastly, gene neighborhood calling was a major time-consuming step of the platform. Therefore, a database scheme for collecting gene neighborhood profile is recommended to improve performance.

## Supplementary information

Supplementary information.
