# Supplementary material for: Photosynthetic protein classification using genome neighborhood-based machine learning feature
Source: Sci Rep. 2020 Apr 28;10:7108. doi: 10.1038/s41598-020-64053-w (PMC7189237; doi:10.1038/s41598-020-64053-w)
Supplement: Supplementary file 1 — Supplementary information. [file 41598_2020_64053_MOESM1_ESM.pdf]

SUPPLEMENTARY DATA

Topic: Photosynthetic protein classification using genome neighborhood-based machine learning feature

Authors: Apiwat Sangphukieo, Teeraphan Laomettachit and Marasri Ruengjitchatchawalya

Text S1 Collection of photosynthetic prokaryote genomes and protein classification protocol

To date, phototropic prokaryotes have been classified into seven phyla (Cyanobacteria, Heliobacteria, Green sulfur bacteria, Green nonsulfur bacteria, Purple bacteria, Acidobacteria, and Gemmatimonadetes) [1]. To collect photosynthetic prokaryote genomes, we manually searched, across the seven phyla, for organisms with reported photosynthetic ability in literature. The complete genomes of 163 photosynthetic prokaryotes, mostly cyanobacteria, were retrieved from the NCBI database. To confirm their photosynthetic ability, homologs of reaction center proteins of the photosynthetic system were investigated. Genomes were considered as containing photosynthetic ability, if they contain at least one of the reaction center homologs (Type I RC: PsaA, PsaB, PshA, PscA, Type II RC: PsbA, PsbB, PufL, PufM) observed by Blastp search (≥50% sequence identity). Eight of the genomes that lacked reaction center genes and the genome of *Roseobacter litoralis* Strain Och 149, whose reaction center genes (*pufL, pufM*) are located in the plasmid [2] were excluded from our analysis. Details about the remaining 154 genomes used in our study are provided in Table S 1. Although genes within the collected genomes have been identified and annotated, different gene finder programs and parameter settings were used, which might introduce differences in the number of identified genes and the quality of annotation. Therefore, we used Prodigal software as part of CMG-biotools workbench [3] to re-identify DNA coding regions and corresponding protein sequences from the genomes. In

order to reduce sequence redundancy, Markov clustering method (MCL) [4] was employed to classify protein sequences into protein families [5].

Protein classification protocol MCL is an efficient tool for classifying protein families, especially with huge datasets and only requires information on sequence similarity relationships, which is generally obtained using Blastp with the all-vs-all protocol. The Blastp all-vs-all comparison was performed on the combined all proteins in a FASTA file that was obtained from a public database or was newly called from genomes by prediction tools. Blastp standalone version was carried out with the NCBI BLAST package v2.2.31. All-vs-all comparison is achieved by using the combined FASTA file searching against database of themselves. Making database of the combined FASTA file is as follows:

makeblastdb -in all_proteins.fasta -parse_seqids -dbtype prot

Blastp was applied with default parameters and output format number 6. The maximum number of HSPs per subject sequence was set to 1. E-value cutoff can be varied to different values (in this study 1E-10, 1E-50 and 1E-100). The command is shown below where [X] is adjusted e-value:

blastp -db all_proteins.fasta -query all_proteins.fasta -out all_proteins.blastout -max_hsps 1 -outfmt '6' -evalue [X] -num_threads 8

MCL version 14-137 was obtained from http://www.micans.org/mcl/. MCL accepts sequence similarity information in 'ABC' format, which is a three-column file containing qseqid, sseqid and e-value. We can simply generate this file via standard Unix command as following.

cut -f 1,2,11 all_proteins.blastout > all_proteins.abc

The abc-format file "all_proteins.abc" is then executed by mcxload, which creates two output files, a network file called "all_proteins.mci" and a label information file in "all_proteins.dict". The

option --stream-mirror option is applied to enforce undirected graph. The option --stream-neg-log10 transform e-value input to log-10 representation. The maximum e-value (1E-200) is set by last option -stream-tf 'ceil(200)'.

mcxload -abc all_proteins.abc -write-tab all_proteins.dict -o all_proteins.mci --stream-mirror --stream-neg-log10 -stream-tf 'ceil(200)'

Now, MCL is ready to run by using all_proteins.mci as input. Only one parameter can be adjusted is inflation values. The higher inflation value yields the higher clustering quality. Output file is automatically created to out.all_proteins.mci.I[Y], where [Y] is inflation value.

mcl all_proteins.mci -I [Y]

Then, the raw output can be converted to a readable format with labels by mcxdump. The output file contains all proteins for a cluster on a single line delimited by tab.

mcxdump -icl out.all_proteins.mci.I[Y] -o dump.all_proteins.mci.I[Y] –tabr all_proteins.dict

Text S 2 Evaluation metrics

We selected and used three different metrics to evaluate the prediction performance of classifiers. Accuracy [6] is the most common metric for classifier evaluation. It measures the overall performance by considering the probability of true value among the predictions.

$$\text{Accuracy} = \frac{TP+TN}{TP+TN+FP+FN}$$

F1 measure [6] is the harmonic mean of precision and recall. Precision is a measurement of correctness i.e. how many examples of the positive class were correctly predicted among the total positive predictions. Recall is a measurement of completeness i.e. how many examples of

the positive class were correctly predicted among the total examples in the positive class. We measure the F1 score of the minor class (photosynthetic class) to measure the real performance of the class-imbalanced dataset.

$$\text{F1 measure} = \frac{2\ x\ Precision\ x\ Recall}{Precision\ x\ Recall}$$

$$\text{Precision} = \frac{TP}{TP + FP}$$

$$\text{Recall} = \frac{TP}{TP + FN}$$

Matthews Correlation Coefficient [7,8] is a single formula measurement metric that considers mutually accuracies and error rates on both classes. It is widely used in bioinformatics area because of its robustness in an imbalanced dataset.

$$\text{MCC} = \frac{(TP\ x\ TN) - (FP\ x\ FN)}{\sqrt{(TP+FP)(TP+FN)(TN+FP)(TN+FN)}}$$

The best achievable score of each classifier after varying threshold is used to be a final score for model comparison.

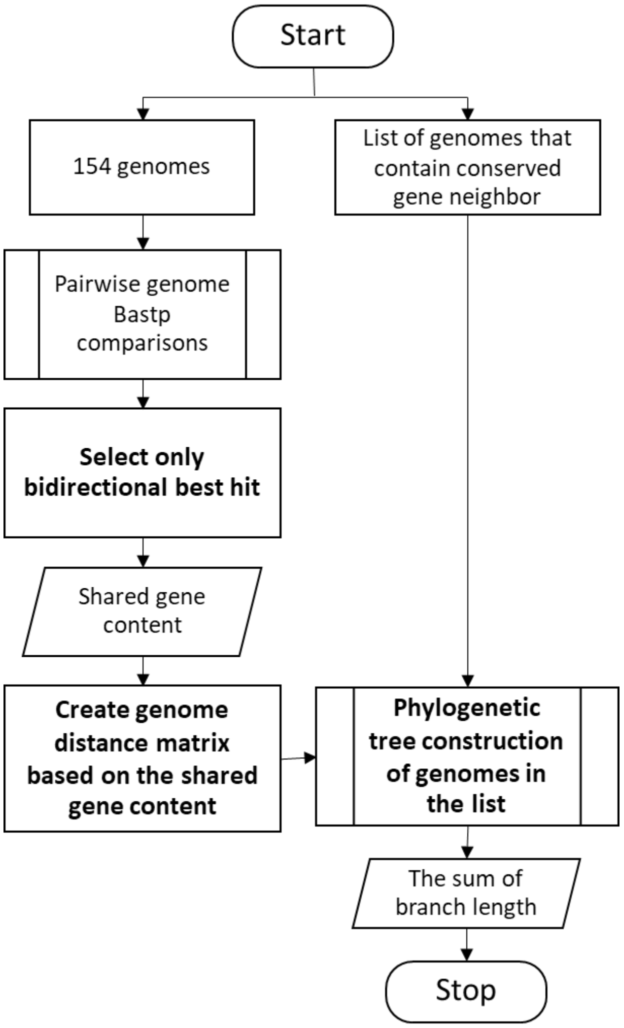

Figure S 1 Flowchart of Phylo score calculation

Complete 154 photosynthetic genomes were retrieved from public databases. Pairwise comparisons of all 154 genomes were performed using Blastp, and the shared gene content of each genome pair was identified by reciprocal best hit, implemented by an in-house python script. The pairwise distance between genomes was calculated by $d = -ln(s)$, where s is the proportion of shared gene content of the two genomes divided by the average number of proteins between the two genomes. The genome distance matrix was constructed based on shared gene content. The phylogenetic tree of the genomes, in which the gene neighbors were identified, was constructed based on the genome distance matrix. The Phylo score was determined by the summation of the total branch length of the tree.

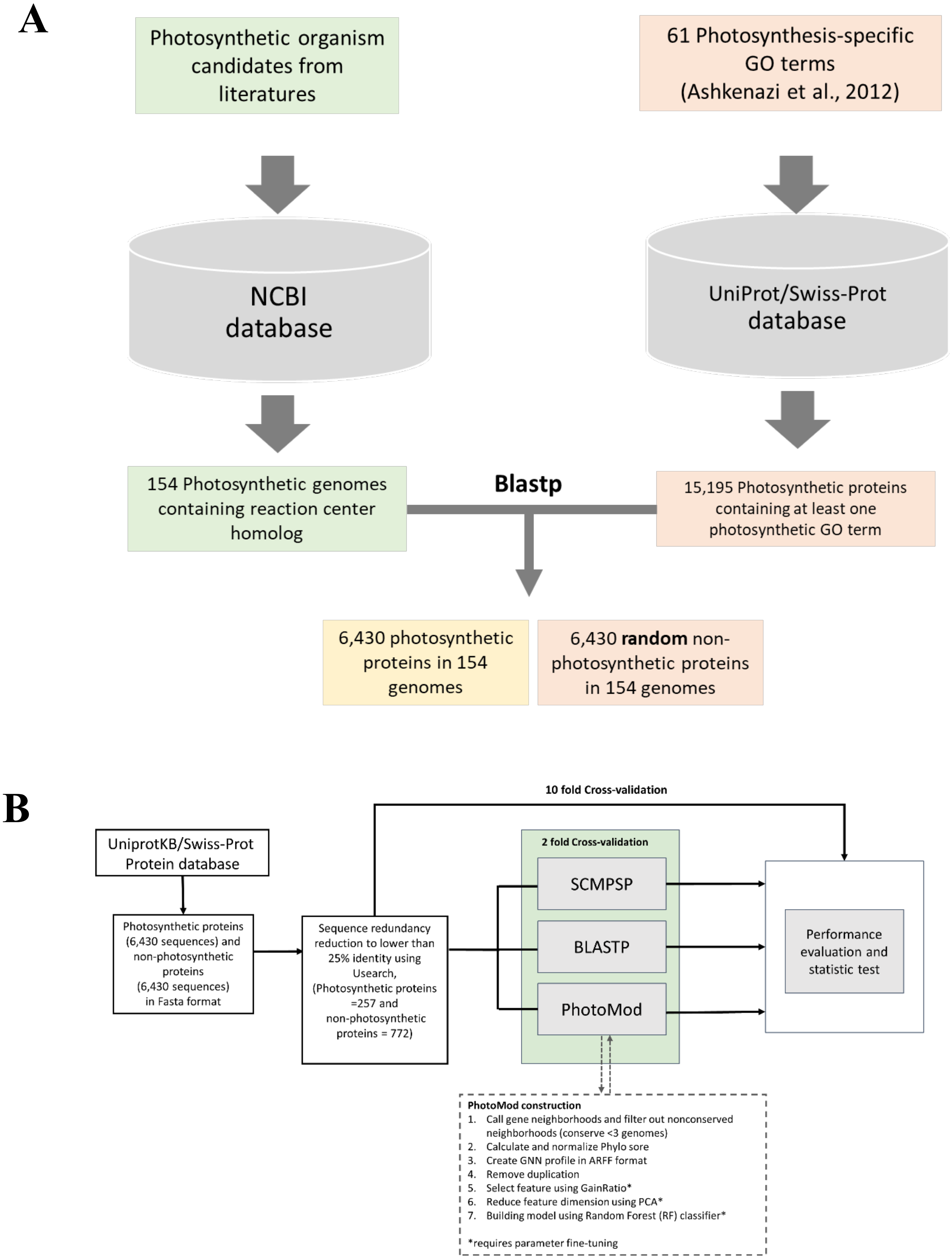
A
Photosynthetic organism candidates from literatures
61 Photosynthesis-specific GO terms (Ashkenazi et al., 2012)
NCBI database
UniProt/Swiss-Prot database
154 Photosynthetic genomes containing reaction center homolog
Blastp
15,195 Photosynthetic proteins containing at least one photosynthetic GO term
6,430 photosynthetic proteins in 154 genomes
6,430 random non-photosynthetic proteins in 154 genomes
B
10 fold Cross-validation
UniprotKB/Swiss-Prot Protein database
Photosynthetic proteins (6,430 sequences) and non-photosynthetic proteins (6,430 sequences) in Fasta format
Sequence redundancy reduction to lower than 25% identity using Usearch, (Photosynthetic proteins =257 and non-photosynthetic proteins = 772)
2 fold Cross-validation
SCMPSP
BLASTP
PhotoMod
Performance evaluation and statistic test
PhotoMod construction
1. Call gene neighborhoods and filter out nonconserved neighborhoods (conserve <3 genomes)
2. Calculate and normalize Phylo sore
3. Create GNN profile in ARFF format
4. Remove duplication
5. Select feature using GainRatio*
6. Reduce feature dimension using PCA*
7. Building model using Random Forest (RF) classifier*
*requires parameter fine-tuning

Figure S 2 The workflow of dataset collection and model performance comparison of PhotoMod to Blastp and SCMPSP

(A) The workflow of dataset collection. Photosynthetic prokaryote candidates selected from literature were used as queries to search for complete genomes in NCBI database. After checking the core reaction center protein homolog, 154 photosynthetic complete genomes were retrieved (see Text S1 for the detail). Photosynthetic proteins were collected from the proteins that contain at least one of the 61 photosynthesis-specific GO terms in UniProtKB/Swiss-Prot database. The photosynthetic proteins were identified in the 154 photosynthetic genomes by Blastp. The non-photosynthetic proteins were randomly selected from the proteins that have no photosynthesis-specific GO term label from the UniProtKB/Swiss-Prot database. (B) The workflow of model performance comparison. The protein datasets were clustered to reduce sequence redundancy to lower than 25% identity before training the models. The two nested 10 folds cross-validation was carried out to evaluate the model performance. The nested fold was used to determine the best parameter set of each model before comparing the model performance of each fold. Wilcoxon signed-rank test was applied to evaluate the differences in the performance of the models. For PhotoMod model construction, an in-house python script was developed to select genome neighborhood. Neighborhoods conserved in < 3 genomes were considered as "noise" and removed. The level of genome neighborhood conservation was calculated as Phylo score. The query proteins with genome neighborhood profiles were transformed into ARFF format, which can be readable by Weka machine learning software. GainRatio and principal component analysis (PCA) were applied to select important features and reduce the dimension of the data, respectively. Random forest (RF) classifier was employed to build the genome neighborhood-based model, PhotoMod.

Chlorobaculum tepidum

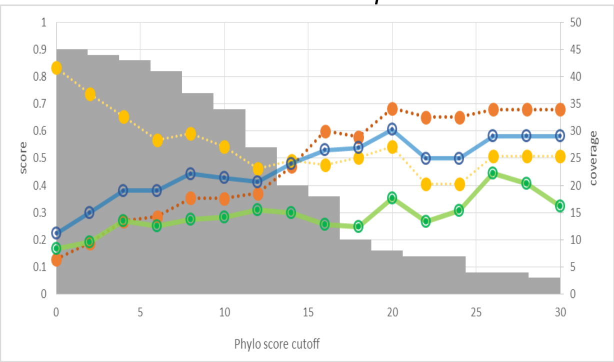

Chloroflexus aurantiacus

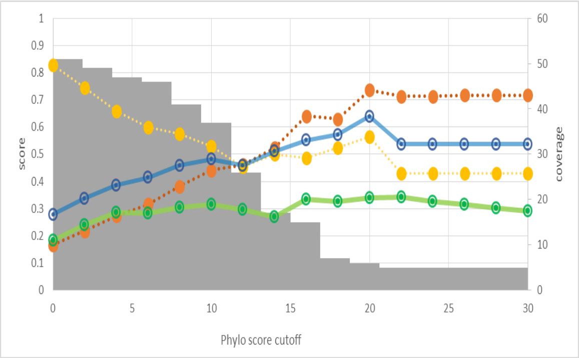

Rhodobacter sphaeroides

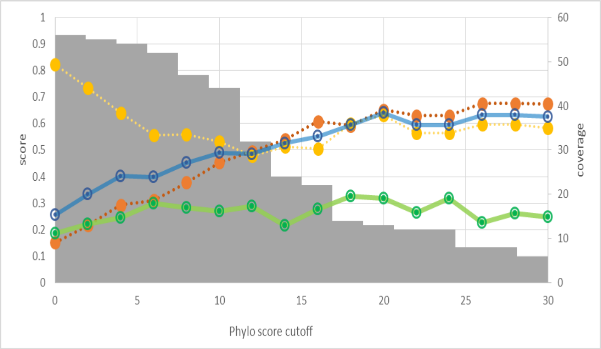

Rhodospirillum rubrum

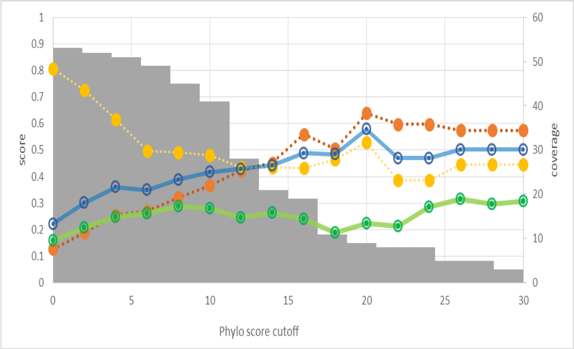

Prochlorococcus marinus

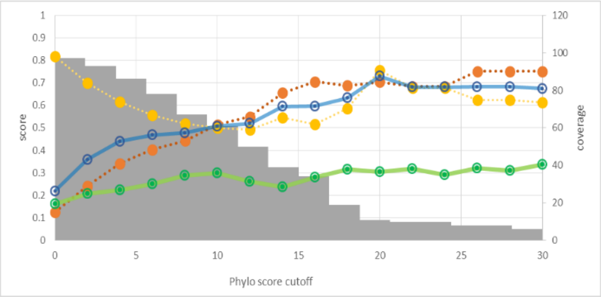

Thermosynechococcus elongatus

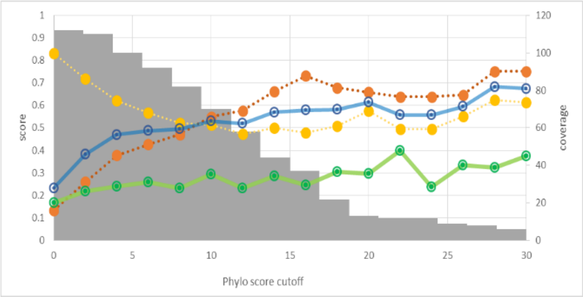

Gloeobacter violaceus

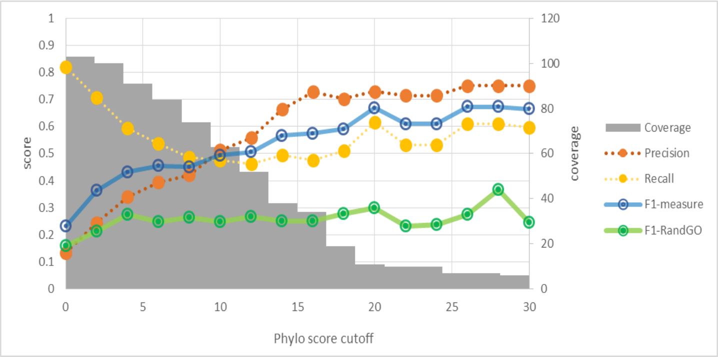

120

Figure S 3 Functional relation measurements between photosynthetic genes and their neighbors using Phylo score as a cutoff criteria

The functional relationship between photosynthetic genes and their neighbors were observed by the similarity of the GO terms shown by F1 measure. The x-axis represents the Phylo score cutoff, while the y-axis represents the F1 measure. The secondary y-axis represents the number of photosynthetic genes containing at least one gene neighbor (coverage). The random GO dataset (RandGO) was sampled with the number equal to the number of GO terms in each conserved neighborhood.

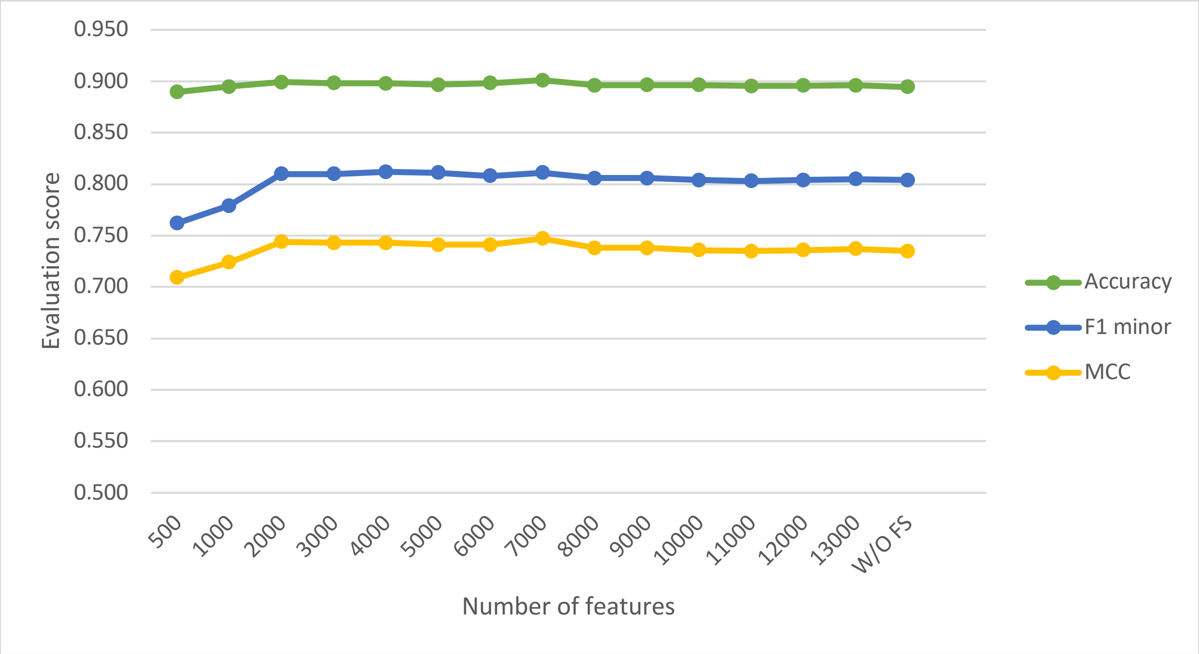

Figure S 4 Minimization of the number of the feature using GainRatio method and random forest classifier

The y-axis represents an evaluation score of the classifier that was constructed from a different number of the selected feature represented by x-axis. The classifier without using the feature selection method is indicated by W/O FS label.

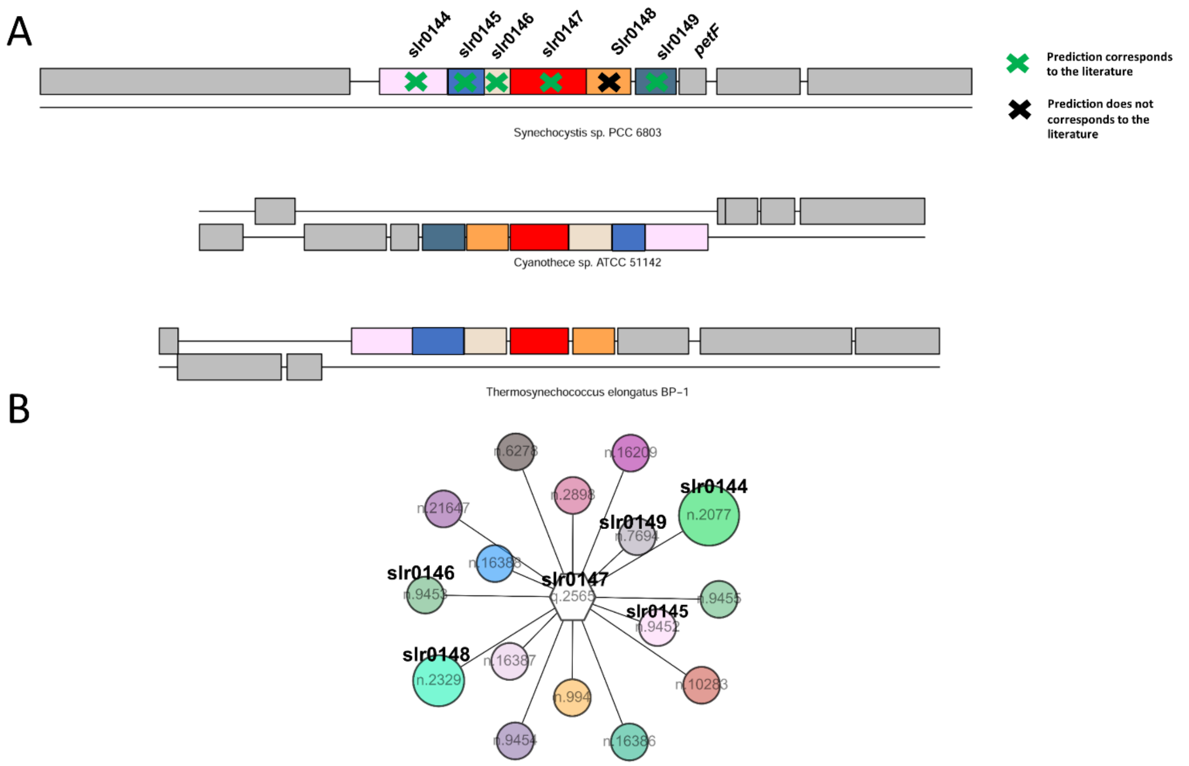

Figure S 5 Illustrating the predictions of unknown proteins from Synechocystis sp. PCC 6803 and genome neighborhood visualization

(A) Visualization of gene neighborhoods of slr0147 in three different genomes. Color in the boxes indicate homologous relationships of the protein-coding genes between genomes, while grey boxes indicate no homologous relationship. Green cross indicates that the prediction result from PhotoMod corresponds with the information from the literature [9], while the black cross indicates that the prediction does not correspond. (B) Genome neighborhood network visualization of slr0147. The slr0147 protein used as a query is represented by a hexagon. The circles represent protein clusters from neighboring genes, the edges of which show gene neighborhood relationship with the query cluster. The size of the circles indicates the conservation corresponding to ‘Phylo score’. The proteins are clustered with E-value cutoff 1E-50. The network indicates that slr0147 mostly prefers to conserve slr0144 as a neighboring gene followed by slr0148, in photosynthetic organisms. The genome neighborhood network was transformed into the genome neighborhood profile and then was used as a feature for the prediction in PhotoMod.

Table S 1 List of 61 photosynthesis-specific GO terms

No.	GO term	Description
1	GO:0015979	Photosynthesis
2	GO:0019684	photosynthesis, light reactions
3	GO:0019685	photosynthesis, dark reactions
4	GO:0080005	photosystem stoichiometry adjustment
5	GO:0009502	photosynthetic electron transport chain
6	GO:0009521	Photosystem
7	GO:0009522	photosystem I
8	GO:0009523	photosystem II
9	GO:0009538	photosystem I reaction center
10	GO:0009539	photosystem II reaction center
11	GO:0009579	photosynthetic membrane
12	GO:0009643	photosynthetic acclimation
13	GO:0009760	C4 photosynthesis
14	GO:0009761	CAM photosynthesis
15	GO:0009762	NADP+malic enzyme C4 photosynthesis
16	GO:0009763	NAD+malic enzyme C4 photosynthesis
17	GO:0009764	PEP carboxykinase C4 photosynthesis
18	GO:0009765	photosynthesis light harvesting
19	GO:0009767	photosynthetic electron transport
20	GO:0009768	photosynthesis light harvesting in photosystem I
21	GO:0009769	photosynthesis light harvesting in photosystem II
22	GO:0009770	primary charge separation in photosystem I
23	GO:0009771	Primary charge separation in photosystem II
24	GO:0009772	photosynthetic electron transport in photosystem II
25	GO:0009773	photosynthetic electron transport in photosystem I
26	GO:0009774	photosynthetic electron transport in plastoquinone
27	GO:0009775	photosynthetic electron transport in cytochrome b6/f
28	GO:0009776	photosynthetic electron transport in plastocyanin
29	GO:0009777	photosynthetic phosphorylation
30	GO:0009778	cyclic photosynthetic phosphorylation
31	GO:0009779	noncyclic photosynthetic phosphorylation
32	GO:0009781	photosynthetic water oxidation
33	GO:0009782	photosystem I antennae complex
34	GO:0009783	photosystem II antennae complex
35	GO:0009854	oxidative photosynthetic carbon pathway
36	GO:0010109	regulation of photosynthesis

37	GO:0010110	regulation of photosynthesis, dark reaction
38	GO:0010206	photosystem II repair
39	GO:0010207	photosystem II assembly
40	GO:0010251	photosystem I assembly
41	GO:0010270	photosystem II oxygen evolving complex assembly
42	GO:0030093	photosystem I (sensu Viridiplantae)
43	GO:0030094	photosystem I (sensu Cyanobacteria)
44	GO:0030095	photosystem II (sensu Viridiplantae)
45	GO:0030096	photosystem II (sensu Cyanobacteria)
46	GO:0034357	photosynthetic membrane
47	GO:0042548	regulation of photosynthesis, light reaction
48	GO:0042549	photosystem II stabilization
49	GO:0042550	photosystem I stabilization
50	GO:0043155	negative regulation of photosynthesis, light reaction
51	GO:0048564	photosystem I assembly
52	GO:0045156	electron transporter, transferring electrons within the cyclic electron transport pathway of photosynthesis
53	GO:0045157	electron transporter, transferring electrons within the non-cyclic electron transport pathway of photosynthesis
54	GO:0045158	transferring electron from †cytochrome b6/f complex in direction to photosystem I
55	GO:0046028	electron transporter, transferring electrons from cytochrome b6/f complex of photosystem II
56	GO:0009507	Chloroplast
57	GO:0044434	chloroplast part
58	GO:0009570	chloroplast stroma
59	GO:0044435	plastid part
60	GO:0009532	plastid stroma
61	GO:0009536	Plastid

Table S 2 List of 154 complete genomes of photosynthetic prokaryotes after filtering by reaction center detection

ID	Taxonomy	Organism	NCBI Accesion
1	Acidobacteria	Candidatus Chloracidobacteriumthermophilum_1	CP002514
1	Acidobacteria	Candidatus Chloracidobacteriumthermophilum_2	CP002515
5	Chlorobi (Green sulfur bacteria)	Chlorobium tepidum	AE006470
6	Chlorobi (Green sulfur bacteria)	Chlorobaculum parvum	CP001099
7	Chlorobi (Green sulfur bacteria)	Chlorobium limicola	CP001097
8	Chlorobi (Green sulfur bacteria)	Chloroherpeton thalassium ATCC 35110	CP001100
9	Chlorobi (Green sulfur bacteria)	Prosthecochloris aestuarii	CP001108
10	Chlorobi (Green sulfur bacteria)	Chlorobium phaeovibrioides DSM 265	CP000607
11	Chlorobi (Green sulfur bacteria)	Chlorobium phaeobacteroides BS1	CP001101
12	Chlorobi (Green sulfur bacteria)	Chlorobium chlorochromatii CaD3	CP000108
13	Chlorobi (Green sulfur bacteria)	Pelodictyon phaeoclathratiforme BU-1	CP001110
14	Chlorobi (Green sulfur bacteria)	Chlorobium luteolum DSM 273	CP000096
15	Chlorobi (Green sulfur bacteria)	Prosthecochloris sp. CIB 2401	CP016432.1
16	Chlorobi (Green sulfur bacteria)	Chlorobium phaeobacteroides DSM 266	CP000492.1
17	Chloroflexi (Green nonsulfur bacteria)	Chloroflexus aurantiacus	CP000909
18	Chloroflexi (Green nonsulfur bacteria)	Roseiflexus castenholzii	CP000804
20	Chloroflexi (Green nonsulfur bacteria)	Roseiflexus sp. RS-1	CP000686
21	Chloroflexi (Green nonsulfur bacteria)	Chloroflexus aggregans DSM 9485	CP001337
22	Chloroflexi (Green nonsulfur bacteria)	Chloroflexus sp. Y-400-fl	CP001364
23	Firmicutes (Heliobacteria)	Heliobacterium modesticaldum Ice1	CP000930
25	Proteobacteria (Purple bacteria)	Allochromatium vinosum	CP001896
26	Proteobacteria (Purple bacteria)	Bradyrhizobium sp. BTAi1	CP000494
27	Proteobacteria (Purple bacteria)	Bradyrhizobium sp. ORS 278	CU234118
28	Proteobacteria (Purple bacteria)	Congregibacter litoralis KT71	CM002299
29	Proteobacteria (Purple bacteria)	Dinoroseobacter shibae DFL 12 = DSM 16493	CP000830
30	Proteobacteria (Purple bacteria)	Jannaschia sp. CCS1	CP000264
34	Proteobacteria (Purple bacteria)	Methylobacterium extorquens PA1	CP000908
35	Proteobacteria (Purple bacteria)	Methylobacterium radiotolerans JCM 2831	CP001001
36	Proteobacteria (Purple bacteria)	Rhodobacter capsulatus	CP001312
37	Proteobacteria (Purple bacteria)	Rhodobacter sphaeroides 2.4.1_1	CP000143
37	Proteobacteria (Purple bacteria)	Rhodobacter sphaeroides 2.4.1_2	CP000144
39	Proteobacteria (Purple bacteria)	Rhodobacter sphaeroides ATCC 17025	CP000661
40	Proteobacteria (Purple bacteria)	Rhodobacter sphaeroides ATCC 17029_1	CP000577
40	Proteobacteria (Purple bacteria)	Rhodobacter sphaeroides ATCC 17029_2	CP000578
42	Proteobacteria (Purple bacteria)	Rhodopseudomonas palustris BisA53	CP000463
43	Proteobacteria (Purple bacteria)	Rhodopseudomonas palustris BisB18	CP000301
44	Proteobacteria (Purple bacteria)	Rhodopseudomonas palustris BisB5	CP000283
45	Proteobacteria (Purple bacteria)	Rhodopseudomonas palustris CGA009	BX571963
46	Proteobacteria (Purple bacteria)	Rhodopseudomonas palustris HaA2	CP000250
47	Proteobacteria (Purple bacteria)	Rhodospirillum rubrum	CP000230
48	Proteobacteria (Purple bacteria)	Roseobacter denitrificans OCh 114	CP000362
50	Proteobacteria (Purple bacteria)	Rubrivivax gelatinosus IL144	AP012320
51	Proteobacteria (Purple bacteria)	Thiocystis violascens	CP003154
52	Proteobacteria (Purple bacteria)	Halorhodospira halophila SL1	CP000544
53	Proteobacteria (Purple bacteria)	Citromicrobium sp. JL477	CP011344.1
55	Proteobacteria (Purple bacteria)	Acidiphilium multivorum AIU301	AP012035
56	Proteobacteria (Purple bacteria)	Bradyrhizobium sp. S23321	AP012279
57	Proteobacteria (Purple bacteria)	Brevundimonas subvibrioides ATCC15264	CP002102
58	Proteobacteria (Purple bacteria)	Methylobacterium chloromethanicum CM4	CP001298
59	Proteobacteria (Purple bacteria)	Methylobacterium extorquens AM1	CP001510
60	Proteobacteria (Purple bacteria)	Methylobacterium extorquens DM4	FP103042

61	Proteobacteria (Purple bacteria)	Methylobacterium populi BJ001	CP001029
62	Proteobacteria (Purple bacteria)	Methylobacterium sp. 4–46	CP000943
63	Proteobacteria (Purple bacteria)	Methylocella silvestris BL2	CP001280
64	Proteobacteria (Purple bacteria)	Rhodobacter sphaeroides KD131	CP001150
65	Proteobacteria (Purple bacteria)	Rhodocista centenaria SW	CP000613
66	Proteobacteria (Purple bacteria)	Rhodomicrobium vannielii ATCC17100	CP002292
67	Proteobacteria (Purple bacteria)	Rhodopseudomonas palustris DX-1	CP002418
68	Proteobacteria (Purple bacteria)	Rhodopseudomonas palustris TIE-1	CP001096
69	Proteobacteria (Purple bacteria)	Rhodospirillum photometricum DSM 122	HE663493
70	Proteobacteria (Purple bacteria)	Rhodospirillum rubrum F11	CP003046
71	Proteobacteria (Purple bacteria)	Marichromatium purpuratum 984	CP007031
72	Gemmatimonadetes	Gemmatimonas phototrophica strain AP64	CP011454
73	Cyanobacteria	Prochlorococcus marinus subsp. marinus str. CCMP1375	AE017126.1
74	Cyanobacteria	Synechococcus elongatus PCC 7942	CP000100.1
75	Cyanobacteria	Microcystis aeruginosa NIES-843	AP009552.1
76	Cyanobacteria	Nostoc punctiforme PCC 73102	CP001037.1
77	Cyanobacteria	Thermosynechococcus elongatus BP-1	BA000039.2
78	Cyanobacteria	Trichodesmium erythraeum IMS101	CP000393.1
79	Cyanobacteria	Gloeobacter violaceus PCC 7421	BA000045.2
80	Cyanobacteria	Acaryochloris marina MBIC11017	CP000828.1
81	Cyanobacteria	Anabaena variabilis ATCC 29413	CP000117.1
82	Cyanobacteria	'Nostoc azollae' 0708	CP002059.1
83	Cyanobacteria	Chroococcidiopsis thermalis PCC 7203	CP003597.1
84	Cyanobacteria	Cyanobacterium stanieri PCC 7202	CP003940.1
85	Cyanobacteria	Anabaena cylindrica PCC 7122	CP003659.1
86	Cyanobacteria	Cyanobium gracile PCC 6307	CP003495.1
87	Cyanobacteria	Dactylococcopsis salina PCC 8305	CP003944.1
88	Cyanobacteria	Oscillatoria acuminata PCC 6304	CP003607.1
89	Cyanobacteria	Crinalium epipsammum PCC 9333	CP003620.1
90	Cyanobacteria	Stanieria cyanosphaera PCC 7437	CP003653.1
91	Cyanobacteria	Synechococcus sp. CC9902	CP000097.1
92	Cyanobacteria	Nostoc sp. PCC 7120	BA000019.2
93	Cyanobacteria	Synechocystis sp. PCC 6803	BA000022.2
94	Cyanobacteria	Calothrix sp. 336/3	CP011382.1
95	Cyanobacteria	Pseudanabaena sp. PCC 7367	CP003592.1
96	Cyanobacteria	Anabaena sp. 90_1	CP003284.1
96	Cyanobacteria	Anabaena sp. 90_2	CP003285.1
98	Cyanobacteria	Geitlerinema sp. PCC 7407	CP003591.1
99	Cyanobacteria	Pleurocapsa sp. PCC 7327	CP003590.1
100	Cyanobacteria	Microcoleus sp. PCC 7113	CP003630.1
101	Cyanobacteria	Gloeocapsa sp. PCC 7428	CP003646.1
102	Cyanobacteria	Cyanothece sp. ATCC 51142_1	CP000806.1
102	Cyanobacteria	Cyanothece sp. ATCC 51142_2	CP000807.1
104	Cyanobacteria	Prochlorococcus sp. MIT 0801	CP007754.1
105	Cyanobacteria	Halothece sp. PCC 7418	CP003945.1
106	Cyanobacteria	Rivularia sp. PCC 7116	CP003549.1
107	Cyanobacteria	Cyanobacterium aponinum PCC 10605	CP003947.1
108	Cyanobacteria	Oscillatoria nigro-viridis PCC 7112	CP003614.1
109	Cyanobacteria	Gloeobacter kilaueensis JS1	CP003587.1
111	Cyanobacteria	Microcystis panniformis FACHB-1757	CP011339.1
112	Cyanobacteria	Geminocystis sp. NIES-3708	AP014815.1
113	Cyanobacteria	Geminocystis sp. NIES-3709	AP014821.1
114	Cyanobacteria	Cyanothece sp. PCC 8801	CP001287.1
115	Cyanobacteria	Fischerella sp. NIES-3754	AP017305.1
116	Cyanobacteria	Calothrix sp. PCC 7507	CP003943.1
117	Cyanobacteria	Nostoc sp. PCC 7524	CP003552.1
118	Cyanobacteria	Synechococcus sp. JA-3-3Ab	CP000239.1
119	Cyanobacteria	Microcystis aeruginosa NIES-2549	CP011304.1
120	Cyanobacteria	Synechococcus elongatus PCC 6301	AP008231.1

121	Cyanobacteria	Prochlorococcus marinus subsp. pastoris str. CCMP1986	BX548174.1
122	Cyanobacteria	Prochlorococcus marinus str. MIT 9313	BX548175.1
123	Cyanobacteria	Synechococcus sp. JA-2-3B'a(2-13)	CP000240.1
124	Cyanobacteria	Nostoc sp. PCC 7107	CP003548.1
125	Cyanobacteria	Synechocystis sp. PCC 6803	AP012205.1
126	Cyanobacteria	Calothrix sp. PCC 6303	CP003610.1
127	Cyanobacteria	Anabaena sp. wa102	CP011456.1
128	Cyanobacteria	Cyanothece sp. PCC 7424	CP001291.1
129	Cyanobacteria	Prochlorococcus sp. MIT 0604	CP007753.1
130	Cyanobacteria	Cyanothece sp. PCC 7425	CP001344.1
131	Cyanobacteria	Synechocystis sp. PCC 6803 substr. GT-I	AP012276.1
132	Cyanobacteria	Nostoc sp. NIES-3756	AP017295.1
133	Cyanobacteria	Synechococcus sp. CC9311	CP000435.1
134	Cyanobacteria	Prochlorococcus marinus str. NATL2A	CP000095.2
135	Cyanobacteria	Prochlorococcus marinus str. MIT 9312	CP000111.1
136	Cyanobacteria	Synechococcus sp. PCC 7002	CP000951.1
137	Cyanobacteria	Leptolyngbya sp. PCC 7376	CP003946.1
138	Cyanobacteria	Synechocystis sp. PCC 6803 substr. PCC-N	AP012277.1
139	Cyanobacteria	Cyanothece sp. PCC 7822	CP002198.1
140	Cyanobacteria	Synechocystis sp. PCC 6803 substr. PCC-P	AP012278.1
141	Cyanobacteria	Prochlorococcus marinus str. AS9601	CP000551.1
142	Cyanobacteria	Prochlorococcus marinus str. MIT 9515	CP000552.1
143	Cyanobacteria	Synechococcus sp. WH 7803	CT971583.1
144	Cyanobacteria	Synechocystis sp. PCC 6803	CP003265.1
145	Cyanobacteria	Cyanothece sp. PCC 8802	CP001701.1
146	Cyanobacteria	Synechocystis sp. PCC 6714	CP007542.1
147	Cyanobacteria	Leptolyngbya sp. O-77	AP017367.1
148	Cyanobacteria	Synechococcus sp. RCC307	CT978603.1
149	Cyanobacteria	Prochlorococcus marinus str. MIT 9301	CP000576.1
150	Cyanobacteria	Prochlorococcus marinus str. MIT 9215	CP000825.1
151	Cyanobacteria	Leptolyngbya sp. NIES-3755	AP017308.1
152	Cyanobacteria	Synechocystis sp. PCC 6803	CP012832.1
153	Cyanobacteria	Prochlorococcus marinus str. NATL1A	CP000553.1
154	Cyanobacteria	Prochlorococcus marinus str. MIT 9303	CP000554.1
155	Cyanobacteria	Synechococcus sp. KORDI-100	CP006269.1
156	Cyanobacteria	Synechococcus sp. KORDI-49	CP006270.1
157	Cyanobacteria	Synechococcus sp. PCC 6312	CP003558.1
158	Cyanobacteria	Synechococcus sp. PCC 7502	CP003594.1
159	Cyanobacteria	Synechococcus sp. CC9605	CP000110.1
160	Cyanobacteria	Synechococcus sp. WH 8109	CP006882.1
161	Cyanobacteria	Synechococcus sp. UTEX 2973	CP006471.1
162	Cyanobacteria	Synechococcus sp. WH 8103	LN847356.1
163	Cyanobacteria	Synechococcus sp. PCC 73109	CP013998.1
164	Cyanobacteria	Synechococcus sp. WH 8102	BX548020.1
165	Cyanobacteria	Microcystis aeruginosa NIES-2481	CP012375.1
166	Cyanobacteria	Synechococcus sp. KORDI-52	CP006271.1
167	Cyanobacteria	Synechococcus sp. PCC 7003	CP016474.1
168	Cyanobacteria	Synechococcus sp. PCC 7117	CP016477.1
169	Cyanobacteria	Synechococcus sp. PCC 8807	CP016483.1

Table S 3 List of seven photosynthetic reference genomes

No.	Organism name	Chromosome ID (GenBank)	Phylum	Photosynthetic protein found (Total = 241)
1	*Chlorobaculum tepidum*	AE006470.1	Chlorobi	63
2	*Chloroflexus aurantiacus*	CP000909.1	Chloroflexi	74
3	*Gloeobacter violaceus*	BA000045.2	Cyanobacteria	178
4	*Prochlorococcus marinus*	AE017126.1	Cyanobacteria	159
5	*Rhodobacter sphaeroides*	CP000144.2,CP000143.2	Proteobacteria	77
6	*Rhodospirillum rubrum*	CP000230.1	Proteobacteria	78
7	*Thermosynechococcus elongatus*	BA000039.2	Cyanobacteria	193

Table S 4 Parameter setting for each classifier

classifier	Parameter setting after fine-tuning	Tuned parameter (range)
BayesNet	Estimator algorithm = SimpleEstimator -A 0.1<br>searchAlgorithm = hill climbing algorithm (K2)	Alpha = 0.1 – 0.9
SMO	calibration method = Logistic<br>kernel = RBFKernel<br>Gamma (-G) = 0.01<br>C parameter (-C) = 10	Gamma = 0.001 - 10<br>C = 0.01 - 1000
RandomForest	numIterations (number of trees) = 100<br>maxDepth = 0 (unlimited)<br>numFeatures (randomly chosen attributes) = 100	Number of tree = 1 - 10000<br>Randomly chosen attributes = 1 - 10000

Table S 5 Random forest classifier performance with different combinations of E-value criteria

E-value criteria	Accuracy	F1 minor	MCC
E10+E50+E100	89.924(1.425)	0.810(0.026)	0.744(0.034)
E10+E50	86.754(1.817)	0.710(0.053)	0.630(0.058)
E10+E100	87.866(2.128)	0.780(0.039)	0.697(0.053)
E50+E100	88.224(1.881)	0.709(0.050)	0.650(0.058)
E10	79.382(2.698)	0.570(0.067)	0.440(0.079)
E50	83.954(2.304)	0.467(0.081)	0.396(0.091)
E100	84.034(2.339)	0.572(0.078)	0.509(0.083)

Table S 6 P-value table of Wilcoxon signed-rank test in the performance comparison of photosynthetic protein classification

	Method	Blastp	SCMPSP
MCC	Blastp	-	-
	SCMPSP	**0.028**	-
	PhotoMod	**0.005**	**0.005**
F1 minor	Blastp	-	-
	SCMPSP	0.721	-
	PhotoMod	**0.005**	**0.005**
Accuracy	Blastp	-	-
	SCMPSP	**0.007**	-
	PhotoMod	**0.009**	**0.005**

*The model performance was observed by nested 10x2 fold cross-validation.

Table S 7 List of 12 novel photosynthetic proteins collected after September 2016

Gene name	Function	Ref
rfpA	control the expression of the Far-red light photoacclimation (FaRLiP) gene cluster	[10]
rfpB	control the expression of the Far-red light photoacclimation (FaRLiP) gene cluster	[10]
IflA	photoreceptor and regulator of cyanobacteriochrome (CBCR) (influenced by far-red light)	[11]
DpxA	photoreceptor and regulator of cyanobacteriochrome (CBCR) (represses phycoerythrin accumulation in yellow light (570–590 nm))	[12]
fciA	transcriptional regulators responsible for tuning the phycourobilin:phycoerythrobilin ratio in response to BL and GL	[13]
fciB	transcriptional regulators responsible for tuning the phycourobilin:phycoerythrobilin ratio in response to BL and GL	[13]
isiX	a specialized antenna protein that function in *Synechococcus* PE, A4 and A14 strains under low irradiance or possibly far-red light (or both) conditions	[14]
apcD4	a specialized antenna protein that function in *Synechococcus* PE, A4 and A14 strains under low irradiance or possibly far-red light (or both) conditions	[14]
apcB3	a specialized antenna protein that function in *Synechococcus* PE, A4 and A14 strains under low irradiance or possibly far-red light (or both) conditions	[14]
MpeZ	a phycoerythrin-specific bilin lyase, contributes to the type IV chromatic acclimation (CA4) response by attaching phycourobilin to C83 within MpeA in blue light	[13]

slr0151	Involve in PSII assembly and repair (tetratricopeptide repeat protein on thylakoid ultrastructure during PS II assembly and repair)	[15]
CyanoP	Involve in the Early Steps of Photosystem II Assembly in the Cyanobacterium	[16]

Table S 8 Novel photosynthetic protein prediction using different methods with varying thresholds

**Method**	**Threshold**	**Accuracy**	**F1 (minor class)**	**MCC**
Blastp	E-value <= 100	0.902	0.000	0.001
	**E-value <= 10**	**0.789**	**0.188**	**0.078**
	E-value <= 1	0.423	0.124	-0.096
	E-value <= 0.1	0.252	0.080	-0.279
	E-value <= 0.01	0.228	0.078	-0.305
	E-value <= 0.001	0.228	0.078	-0.305
SVMProt*	**Probability >= 50%**	**0.650**	**0.218**	**0.104**
	Probability >= 60%	0.846	0.174	0.089
	Probability >= 70%	0.829	0.087	-0.007
	Probability >= 80%	0.772	0.067	-0.059
	Probability >= 90%	0.675	0.000	-0.179
PhotoMod	E-value <= 10	0.789	0.235	0.133
	**E-value <= 1**	**0.772**	**0.300**	**0.214**
	E-value <= 0.1	0.748	0.279	0.188
	E-value <= 0.01	0.659	0.222	0.110
	E-value <= 0.001	0.220	0.094	-0.269

*True positive is counted if predicted GO terms of positive input contain at least one of 61 photosynthetic GO terms, whereas false positive is counted if at least one of 61 photosynthetic GO terms is found in predicted GO terms of negative input.
[a]Although the probability threshold of DeepGO was adjusted to the lowest, we could not detect the true-positive.
[b]Note that SCMPSP prediction result does not provide the prediction score or probability score.

Table S 9 The result of four methods in the prediction of novel photosynthetic proteins

No	Protein	Actual class	Blastp	SVMProt	SCMPSP	PHOTOMOD
1	apcB3	photo	photo	photo	photo	photo
2	apcD4	photo	photo	non_photo	non_photo	photo
3	CyanoP	photo	non_photo	photo	photo	non_photo
4	DpxA	photo	non_photo	non_photo	non_photo	non_photo
5	fciA	photo	non_photo	photo	non_photo	photo
6	fciB	photo	non_photo	non_photo	non_photo	photo
7	IflA	photo	non_photo	non_photo	non_photo	non_photo
8	isiX	photo	non_photo	photo	photo	photo
9	MpeZ	photo	non_photo	photo	non_photo	photo
10	rfpA	photo	non_photo	non_photo	non_photo	non_photo
11	rfpB	photo	photo	photo	non_photo	non_photo
12	slr0151	photo	non_photo	non_photo	non_photo	non_photo
13	A0A0D4BS77	non_photo	non_photo	non_photo	non_photo	non_photo
14	A0A0D4BSN8	non_photo	non_photo	non_photo	non_photo	non_photo
15	A0A0H3AJC2	non_photo	non_photo	non_photo	non_photo	non_photo
16	A0A0H3AKU6	non_photo	non_photo	non_photo	photo	non_photo
17	A0A0S3QTC6	non_photo	non_photo	photo	non_photo	non_photo
18	A0A0S3QTD0	non_photo	non_photo	non_photo	non_photo	photo
19	A0A1E7MYN1	non_photo	non_photo	photo	non_photo	non_photo
20	A0QTU7	non_photo	non_photo	photo	non_photo	non_photo
21	A0QTV0	non_photo	non_photo	photo	photo	non_photo
22	A0R3Y2	non_photo	non_photo	non_photo	non_photo	non_photo
23	A7AZH2	non_photo	non_photo	non_photo	non_photo	non_photo
24	A7B3K3	non_photo	non_photo	non_photo	non_photo	non_photo
25	A7NH01	non_photo	non_photo	non_photo	non_photo	photo
26	A9AWD5	non_photo	non_photo	non_photo	non_photo	non_photo
27	A9AWD6	non_photo	non_photo	non_photo	non_photo	NotPredict
28	A9AWD7	non_photo	photo	non_photo	non_photo	non_photo
29	A9FZ87	non_photo	non_photo	non_photo	non_photo	photo
30	B1VXR4	non_photo	non_photo	non_photo	non_photo	non_photo
31	B1W3T1	non_photo	photo	non_photo	non_photo	non_photo
32	B2FI29	non_photo	non_photo	non_photo	photo	non_photo
33	B5H7H3	non_photo	photo	non_photo	photo	photo
34	B5HDJ6	non_photo	non_photo	non_photo	non_photo	photo
35	B7J3C9	non_photo	non_photo	photo	photo	non_photo
36	C7PLV2	non_photo	non_photo	non_photo	photo	photo
37	C8WGQ3	non_photo	non_photo	photo	non_photo	non_photo
38	C8WJW0	non_photo	non_photo	photo	non_photo	non_photo
39	C8WMP0	non_photo	non_photo	non_photo	non_photo	non_photo
40	D2B747	non_photo	photo	photo	photo	photo
41	D2PPM7	non_photo	non_photo	non_photo	non_photo	non_photo

42	D2PPM8	non_photo	non_photo	photo	non_photo	photo
43	D8GR66	non_photo	non_photo	photo	non_photo	non_photo
44	D8GR67	non_photo	photo	photo	photo	non_photo
45	D8GR68	non_photo	non_photo	non_photo	photo	non_photo
46	D8GR69	non_photo	photo	non_photo	photo	non_photo
47	D8GR70	non_photo	non_photo	non_photo	photo	non_photo
48	D8GR71	non_photo	photo	non_photo	non_photo	non_photo
49	D9XD61	non_photo	non_photo	non_photo	non_photo	photo
50	D9XDR8	non_photo	non_photo	non_photo	non_photo	photo
51	E4N7E5	non_photo	non_photo	photo	non_photo	photo
52	E8W6C7	non_photo	non_photo	photo	non_photo	photo
53	E9RFT0	non_photo	non_photo	non_photo	non_photo	non_photo
54	F9US27	non_photo	non_photo	photo	non_photo	non_photo
55	F9UT67	non_photo	non_photo	non_photo	non_photo	photo
56	F9UT68	non_photo	non_photo	photo	non_photo	non_photo
57	H2A7G5	non_photo	non_photo	non_photo	non_photo	NotPredict
58	H2K885	non_photo	non_photo	non_photo	non_photo	non_photo
59	H2K888	non_photo	non_photo	non_photo	non_photo	non_photo
60	H6LC27	non_photo	non_photo	photo	non_photo	non_photo
61	H6LC29	non_photo	non_photo	NotPredict	photo	non_photo
62	H6LC30	non_photo	photo	non_photo	photo	non_photo
63	H6LC31	non_photo	photo	non_photo	photo	non_photo
64	H6LC32	non_photo	non_photo	non_photo	non_photo	non_photo
65	K0K750	non_photo	non_photo	non_photo	non_photo	photo
66	K4JY29	non_photo	photo	NotPredict	photo	NotPredict
67	K4REQ6	non_photo	non_photo	non_photo	photo	photo
68	L8EUQ6	non_photo	non_photo	photo	non_photo	non_photo
69	L8EYU3	non_photo	non_photo	non_photo	non_photo	non_photo
70	O34138	non_photo	non_photo	non_photo	non_photo	non_photo
71	O51767	non_photo	non_photo	non_photo	non_photo	non_photo
72	O54143	non_photo	non_photo	non_photo	photo	non_photo
73	O83323	non_photo	non_photo	non_photo	photo	non_photo
74	O83324	non_photo	non_photo	non_photo	photo	non_photo
75	P0DPE4	non_photo	non_photo	photo	non_photo	non_photo
76	P0DPE9	non_photo	photo	photo	photo	non_photo
77	P0DPF0	non_photo	non_photo	non_photo	non_photo	non_photo
78	P71889	non_photo	non_photo	non_photo	non_photo	non_photo
79	P96072	non_photo	non_photo	photo	non_photo	non_photo
80	Q0P9Y2	non_photo	non_photo	non_photo	photo	photo
81	Q0SJK9	non_photo	non_photo	non_photo	non_photo	non_photo
82	Q0ZQ46	non_photo	non_photo	non_photo	non_photo	non_photo
83	Q15JF5	non_photo	non_photo	photo	photo	non_photo
84	Q15JF8	non_photo	non_photo	photo	non_photo	non_photo
85	Q2PWU9	non_photo	non_photo	non_photo	non_photo	non_photo

86	Q31KC7	non_photo	non_photo	NotPredict	non_photo	photo
87	Q3K999	non_photo	non_photo	NotPredict	non_photo	non_photo
88	Q3T6E2	non_photo	non_photo	non_photo	non_photo	non_photo
89	Q46085	non_photo	non_photo	NotPredict	non_photo	non_photo
90	Q4KCY6	non_photo	non_photo	photo	non_photo	non_photo
91	Q4VKU9	non_photo	non_photo	non_photo	non_photo	non_photo
92	Q4VKV0	non_photo	non_photo	photo	non_photo	non_photo
93	Q4VKV1	non_photo	photo	photo	photo	non_photo
94	Q5SHW0	non_photo	non_photo	non_photo	photo	non_photo
95	Q5SIP0	non_photo	non_photo	non_photo	non_photo	non_photo
96	Q65YW9	non_photo	non_photo	photo	non_photo	non_photo
97	Q65YX0	non_photo	non_photo	non_photo	photo	non_photo
98	Q6EZC2	non_photo	non_photo	non_photo	non_photo	non_photo
99	Q6EZC3	non_photo	non_photo	non_photo	photo	non_photo
100	Q72EF3	non_photo	non_photo	photo	non_photo	photo
101	Q72EF4	non_photo	non_photo	non_photo	non_photo	non_photo
102	Q768S8	non_photo	non_photo	non_photo	non_photo	non_photo
103	Q768T3	non_photo	non_photo	non_photo	non_photo	non_photo
104	Q7N561	non_photo	non_photo	non_photo	non_photo	photo
105	Q81L64	non_photo	photo	non_photo	photo	non_photo
106	Q81L65	non_photo	non_photo	photo	non_photo	non_photo
107	Q81LM1	non_photo	non_photo	non_photo	non_photo	non_photo
108	Q81QL7	non_photo	non_photo	non_photo	photo	non_photo
109	Q81XB1	non_photo	non_photo	non_photo	photo	non_photo
110	Q81XB2	non_photo	NotPredict	non_photo	photo	non_photo
111	Q81XB3	non_photo	non_photo	photo	non_photo	non_photo
112	Q826W3	non_photo	non_photo	non_photo	non_photo	non_photo
113	Q845S8	non_photo	non_photo	non_photo	photo	non_photo
114	Q845S9	non_photo	non_photo	non_photo	non_photo	non_photo
115	Q899Y1	non_photo	non_photo	photo	photo	non_photo
116	Q8A712	non_photo	non_photo	non_photo	non_photo	non_photo
117	Q8GHB1	non_photo	non_photo	non_photo	non_photo	non_photo
118	Q8PHA1	non_photo	non_photo	non_photo	non_photo	non_photo
119	Q9AF95	non_photo	photo	non_photo	photo	non_photo
120	Q9AGW3	non_photo	photo	photo	photo	non_photo
121	Q9LCB4	non_photo	non_photo	non_photo	non_photo	non_photo
122	Q9X721	non_photo	photo	NotPredict	photo	non_photo
123	S4S3E3	non_photo	non_photo	non_photo	non_photo	non_photo

*Green color is used to emphasize the correct prediction, while red color is used to emphasize incorrect prediction.

190

Table S 10 PhotoMod prediction result of unknown proteins in cyanobacteria genome, *Synechocystis* sp. PCC 6803

Of the 1,885 unknown protein-coding genes in *Synechocystis* sp. PCC 6803, 479 sequences (~26%) were predicted to be involved in photosynthesis. BlastHits is the number of proteins in our protein database (collected from photosynthetic organisms) that match the query. PhotoHits(%) is the proportion of matched proteins that were predicted as photosynthetic protein, whereas nonPhotoHits(%) is the proportion of matched proteins that are predicted as nonphotosynthetic protein.NoPredictHits is the number of BlastHits that could not be predicted by PhotoMod. Photosynthesis class (photo) is assigned if the PhotoHits is higher than 50%. The average prediction probability of PhotoHits is shown.

Query	BlastHits	PhotoHits(%)	nonPhotoHits(%)	NoPredictHits	Prediction Class	Average probability
sgl0002	33	57.576	42.424	0	photo	0.683
sll0037	143	52.448	47.552	0	photo	0.666
sll0069	78	96.154	3.846	0	photo	0.698
sll0088	138	78.261	21.739	0	photo	0.941
sll0149	60	96.667	3.333	0	photo	0.562
sll0160	64	92.187	7.812	0	photo	0.806
sll0178	24	54.167	45.833	0	photo	0.682
sll0185	126	100.000	0.000	0	photo	0.961
sll0253	36	97.222	2.778	0	photo	0.663
sll0272	94	100.000	0.000	0	photo	0.822
sll0318	146	100.000	0.000	0	photo	0.734
sll0364	111	67.568	32.432	0	photo	0.760
sll0413	136	93.382	6.618	0	photo	0.553
sll0423	48	93.750	6.250	0	photo	0.672
sll0436	66	100.000	0.000	0	photo	0.915
sll0442	45	93.333	6.667	0	photo	0.673
sll0471	162	87.037	12.963	0	photo	0.680
sll0497	91	97.802	2.198	0	photo	0.658
sll0528	263	55.133	44.867	0	photo	0.671
sll0543	18	88.889	11.111	0	photo	0.750
sll0544	121	80.992	19.008	0	photo	0.587
sll0572	14	92.857	7.143	0	photo	0.669
sll0585	107	86.916	13.084	0	photo	0.778
sll0611	60	98.333	1.667	0	photo	0.897
sll0615	208	69.712	30.288	0	photo	0.852
sll0639	56	92.857	7.143	0	photo	0.670
sll0696	160	56.250	43.750	0	photo	0.639
sll0732	17	88.235	11.765	0	photo	0.673
sll0814	73	100.000	0.000	0	photo	0.741
sll0853	203	60.591	39.409	0	photo	0.795
sll0860	98	71.429	28.571	0	photo	0.665
sll0861	109	88.991	11.009	0	photo	0.574
sll0997	38	94.737	5.263	0	photo	0.554
sll1060	90	98.889	1.111	0	photo	0.777
sll1158	27	100.000	0.000	0	photo	0.654
sll1242	558	68.638	31.362	0	photo	0.641
sll1340	93	98.925	1.075	0	photo	0.940
sll1372	98	69.388	30.612	0	photo	0.558
sll1381	43	86.047	13.953	0	photo	0.550
sll1389	24	100.000	0.000	0	photo	0.700
sll1390	96	82.292	17.708	0	photo	0.683
sll1399	114	100.000	0.000	0	photo	0.842
sll1400	106	69.811	30.189	0	photo	0.817
sll1414	94	100.000	0.000	0	photo	0.987
sll1455	84	100.000	0.000	0	photo	0.654
sll1485	129	99.225	0.775	0	photo	0.730
sll1486	127	99.213	0.787	0	photo	0.732
sll1500	95	98.947	1.053	0	photo	0.584
sll1532	57	100.000	0.000	0	photo	0.654
sll1543	52	94.231	5.769	0	photo	0.658
sll1573	56	94.643	5.357	0	photo	0.847
sll1608	106	88.679	11.321	0	photo	0.538

sll1738	192	58.333	41.667	0	photo	0.735
sll1757	95	96.842	3.158	0	photo	0.828
sll1874	190	99.474	0.526	0	photo	0.934
sll1913	279	55.556	44.444	0	photo	0.742
sll1915	76	89.474	10.526	0	photo	0.742
sll1916	137	64.964	35.036	0	photo	0.867
sll1940	107	73.832	26.168	0	photo	0.712
sll1979	97	92.784	7.216	0	photo	0.869
sll5003	124	71.774	28.226	0	photo	0.812
sll5030	10	100.000	0.000	0	photo	0.654
sll5032	98	86.735	13.265	0	photo	0.613
sll5033	80	100.000	0.000	0	photo	0.701
sll5034	49	55.102	44.898	0	photo	0.676
sll5097	89	91.011	8.989	0	photo	0.664
sll5130	6	100.000	0.000	0	photo	0.654
sll5132	54	96.296	3.704	0	photo	0.654
sll6054	7	57.143	42.857	0	photo	0.659
sll6055	10	70.000	30.000	0	photo	0.738
sll7031	59	98.305	1.695	0	photo	0.656
sll7033	112	60.714	39.286	0	photo	0.754
sll7069	15	100.000	0.000	0	photo	0.654
sll8004	39	69.231	30.769	0	photo	0.696
sll8018	4	100.000	0.000	0	photo	0.654
sll8019	5	80.000	20.000	0	photo	0.717
sll8025	22	95.455	4.545	0	photo	0.650
sll8032	5	100.000	0.000	0	photo	0.654
sll8035	8	100.000	0.000	0	photo	0.654
slr0022	94	97.872	2.128	0	photo	0.867
slr0076	290	99.655	0.345	0	photo	0.988
slr0142	37	51.351	48.649	0	photo	0.686
**slr0144**	112	98.214	1.786	0	photo	0.768
**slr0146**	67	98.507	1.493	0	photo	0.710
**slr0147**	93	96.774	3.226	0	photo	0.745
**slr0148**	293	63.823	36.177	0	photo	0.741
**slr0149**	250	97.600	2.400	0	photo	0.914
slr0211	89	77.528	22.472	0	photo	0.653
slr0249	95	100.000	0.000	0	photo	0.821
slr0263	54	98.148	1.852	0	photo	0.616
slr0299	8	100.000	0.000	0	photo	0.563
slr0320	606	57.591	42.409	0	photo	0.703
slr0356	139	74.101	25.899	0	photo	0.763
slr0363	93	95.699	4.301	0	photo	0.660
slr0388	138	99.275	0.725	0	photo	0.858
slr0397	62	100.000	0.000	0	photo	0.544
slr0404	107	73.832	26.168	0	photo	0.553
slr0438	89	100.000	0.000	0	photo	0.553
slr0554	99	93.939	6.061	0	photo	0.665
slr0598	94	98.936	1.064	0	photo	0.864
slr0619	65	60.000	40.000	0	photo	0.769
slr0625	43	97.674	2.326	0	photo	0.679
slr0642	78	88.462	11.538	0	photo	0.687
slr0725	28	100.000	0.000	0	photo	0.654
slr0732	90	100.000	0.000	0	photo	0.651
slr0770	392	50.255	49.745	0	photo	0.830
slr0771	157	67.516	32.484	0	photo	0.735
slr0780	136	58.824	41.176	0	photo	0.656
slr0863	457	94.967	5.033	0	photo	0.664
slr0869	76	78.947	21.053	0	photo	0.818
slr0975	65	100.000	0.000	0	photo	0.654
slr1100	32	100.000	0.000	0	photo	0.893
slr1170	241	71.078	28.922	37	photo	0.854
slr1173	53	94.340	5.660	0	photo	0.656
slr1174	95	87.368	12.632	0	photo	0.655
slr1182	115	75.652	24.348	0	photo	0.702
slr1186	50	98.000	2.000	0	photo	0.808
slr1188	165	97.576	2.424	0	photo	0.741
slr1195	95	96.842	3.158	0	photo	0.861
slr1220	116	81.034	18.966	0	photo	0.850
slr1260	80	100.000	0.000	0	photo	0.701

slr1261	501	51.209	48.791	46	photo	0.691
slr1266	125	84.000	16.000	0	photo	0.754
slr1353	120	91.667	8.333	0	photo	0.659
slr1394	94	98.936	1.064	0	photo	0.861
slr1413	128	78.906	21.094	0	photo	0.725
slr1451	69	89.855	10.145	0	photo	0.782
slr1478	49	91.837	8.163	0	photo	0.662
slr1495	107	85.981	14.019	0	photo	0.948
slr1546	79	92.405	7.595	0	photo	0.633
slr1570	100	68.000	32.000	0	photo	0.817
slr1572	95	71.579	28.421	0	photo	0.815
slr1601	71	95.775	4.225	0	photo	0.947
slr1624	82	82.927	17.073	0	photo	0.672
slr1676	140	60.714	39.286	0	photo	0.638
slr1699	118	98.305	1.695	0	photo	0.911
slr1732	69	100.000	0.000	0	photo	0.845
slr1767	104	71.154	28.846	0	photo	0.844
slr1770	63	96.825	3.175	0	photo	0.654
slr1799	149	75.168	24.832	0	photo	0.594
slr1800	84	97.619	2.381	0	photo	0.769
slr1880	54	90.741	9.259	0	photo	0.663
slr1885	50	98.000	2.000	0	photo	0.579
slr1907	57	100.000	0.000	0	photo	0.654
slr1923	114	94.737	5.263	0	photo	0.517
slr1927	130	54.615	45.385	0	photo	0.745
slr1949	144	99.306	0.694	0	photo	0.741
slr1998	391	65.473	34.527	0	photo	0.796
slr2000	110	67.273	32.727	0	photo	0.662
slr2003	37	100.000	0.000	0	photo	0.645
slr2025	64	73.437	26.562	0	photo	0.524
slr2052	44	97.727	2.273	0	photo	0.655
slr2070	51	86.275	13.725	0	photo	0.815
slr5012	16	100.000	0.000	0	photo	0.654
slr5087	9	77.778	22.222	0	photo	0.689
slr5101	11	100.000	0.000	0	photo	0.654
slr5102	12	100.000	0.000	0	photo	0.654
slr6029	6	83.333	16.667	0	photo	0.699
slr6049	22	59.091	40.909	0	photo	0.647
slr6051	3	100.000	0.000	0	photo	0.654
slr6057	9	66.667	33.333	0	photo	0.700
slr6088	6	83.333	16.667	0	photo	0.699
slr6094	41	53.659	46.341	0	photo	0.722
slr6104	7	85.714	14.286	0	photo	0.664
slr6106	32	100.000	0.000	0	photo	0.654
slr7012	36	100.000	0.000	0	photo	0.654
slr7013	34	100.000	0.000	0	photo	0.654
slr7023	34	76.471	23.529	0	photo	0.669
slr7024	65	87.692	12.308	0	photo	0.644
slr7037	93	95.699	4.301	0	photo	0.661
slr7058	35	54.286	45.714	0	photo	0.727
slr7059	15	66.667	33.333	0	photo	0.719
slr7076	3	100.000	0.000	0	photo	0.669
slr7091	16	93.750	6.250	0	photo	0.659
slr7094	10	100.000	0.000	0	photo	0.654
slr7096	12	100.000	0.000	0	photo	0.690
slr7097	33	100.000	0.000	0	photo	0.654
smr0015	32	84.375	15.625	0	photo	0.720
ssl0241	88	72.727	27.273	0	photo	0.687
ssl0294	60	95.000	5.000	0	photo	0.660
ssl0312	53	100.000	0.000	0	photo	0.654
ssl0385	110	77.273	22.727	0	photo	0.714
ssl0483	49	67.347	32.653	0	photo	0.836
ssl0511	30	96.667	3.333	0	photo	0.664
ssl0788	69	94.203	5.797	0	photo	0.570
ssl0900	11	81.818	18.182	0	photo	0.674
ssl1004	147	87.075	12.925	0	photo	0.660
ssl1046	13	92.308	7.692	0	photo	0.656
ssl1376	177	50.847	49.153	0	photo	0.770
ssl1378	64	95.312	4.687	0	photo	0.557

ssl2009	94	79.787	20.213	0	photo	0.722
ssl2648	81	51.852	48.148	0	photo	0.763
ssl2717	66	95.455	4.545	0	photo	0.925
ssl2920	70	100.000	0.000	0	photo	0.940
ssl2921	130	71.538	28.462	0	photo	0.815
ssl2999	24	95.833	4.167	0	photo	0.663
ssl3297	26	100.000	0.000	0	photo	0.507
ssl3719	126	50.794	49.206	0	photo	0.711
ssl3803	26	84.615	15.385	0	photo	0.647
ssl5031	85	97.647	2.353	0	photo	0.614
ssl5095	33	96.970	3.030	0	photo	0.658
ssl5099	38	55.263	44.737	0	photo	0.710
ssl7042	2	100.000	0.000	0	photo	0.654
ssl7048	15	100.000	0.000	0	photo	0.654
ssl7053	4	100.000	0.000	0	photo	0.899
ssl8028	16	100.000	0.000	0	photo	0.654
ssr0755	66	57.576	42.424	0	photo	0.708
ssr1558	237	75.527	24.473	0	photo	0.858
ssr1698	109	77.982	22.018	0	photo	0.866
ssr1880	61	95.082	4.918	0	photo	0.782
ssr1951	59	100.000	0.000	0	photo	0.637
ssr2009	77	50.649	49.351	0	photo	0.769
ssr2062	71	94.366	5.634	0	photo	0.671
ssr2781	64	100.000	0.000	0	photo	0.905
ssr2806	69	79.710	20.290	0	photo	0.677
ssr3189	87	63.218	36.782	0	photo	0.718
ssr3304	255	66.667	33.333	0	photo	0.905
ssr5011	81	100.000	0.000	0	photo	0.925
ssr5019	249	99.197	0.803	0	photo	0.561
ssr5106	15	86.667	13.333	0	photo	0.686
ssr6046	15	86.667	13.333	0	photo	0.639
ssr8013	35	68.571	31.429	0	photo	0.664
slr1667	12	91.667	8.333	0	photo	0.678
slr9003	88	95.455	4.545	0	photo	0.654
ssr9004	5	100.000	0.000	0	photo	0.654
sll0047	69	100.000	0.000	0	photo	0.883
sll1509	109	99.083	0.917	0	photo	0.889
ssl1417	92	98.913	1.087	0	photo	0.548
sll0584	91	82.418	17.582	0	photo	0.636
slr0480	856	55.023	44.977	0	photo	0.755
sll1214	189	100.000	0.000	0	photo	0.935
slr0923	94	97.872	2.128	0	photo	0.659
sll0101	35	74.286	25.714	0	photo	0.675
sll0167	11	100.000	0.000	0	photo	0.579
sll0225	33	93.939	6.061	0	photo	0.884
sll0263	21	85.714	14.286	0	photo	0.911
sll0265	22	90.909	9.091	0	photo	0.879
sll0266	20	100.000	0.000	0	photo	0.903
sll0280	54	87.037	12.963	0	photo	0.657
sll0327	25	60.000	40.000	0	photo	0.734
sll0328	15	93.333	6.667	0	photo	0.835
sll0403	8	100.000	0.000	0	photo	0.654
sll0405	9	88.889	11.111	0	photo	0.688
sll0406	17	64.706	35.294	0	photo	0.669
sll0419	7	100.000	0.000	0	photo	0.654
sll0426	10	100.000	0.000	0	photo	0.654
sll0443	13	100.000	0.000	0	photo	0.654
sll0444	9	100.000	0.000	0	photo	0.654
sll0445	22	68.182	31.818	0	photo	0.710
sll0446	23	78.261	21.739	0	photo	0.707
sll0447	19	94.737	5.263	0	photo	0.659
sll0448	20	95.000	5.000	0	photo	0.657
sll0449	16	100.000	0.000	0	photo	0.654
sll0494	8	100.000	0.000	0	photo	0.654
sll0508	9	100.000	0.000	0	photo	0.654
sll0539	11	81.818	18.182	0	photo	0.837
sll0552	10	90.000	10.000	0	photo	0.764
sll0563	17	100.000	0.000	0	photo	0.654
sll0588	14	100.000	0.000	0	photo	0.678

sll0595	41	51.220	48.780	0	photo	0.685
sll0614	18	100.000	0.000	0	photo	0.584
sll0623	56	100.000	0.000	0	photo	0.864
sll0625	18	83.333	16.667	0	photo	0.674
sll0630	12	100.000	0.000	0	photo	0.654
sll0722	9	88.889	11.111	0	photo	0.683
sll0733	36	97.222	2.778	0	photo	0.657
sll0847	95	67.368	32.632	0	photo	0.668
sll0872	18	55.556	44.444	0	photo	0.756
sll0943	16	93.750	6.250	0	photo	0.659
sll0980	28	82.143	17.857	0	photo	0.659
sll0981	12	75.000	25.000	0	photo	0.698
sll1061	21	90.476	9.524	0	photo	0.663
sll1062	19	100.000	0.000	0	photo	0.654
sll1086	60	100.000	0.000	0	photo	0.654
sll1132	15	100.000	0.000	0	photo	0.654
sll1163	40	57.500	42.500	0	photo	0.706
sll1239	15	53.333	46.667	0	photo	0.781
sll1240	8	100.000	0.000	0	photo	0.654
sll1241	9	88.889	11.111	0	photo	0.679
sll1267	13	92.308	7.692	0	photo	0.670
sll1268	74	95.946	4.054	0	photo	0.948
sll1272	11	100.000	0.000	0	photo	0.654
sll1273	20	90.000	10.000	0	photo	0.664
sll1338	23	100.000	0.000	0	photo	0.654
sll1359	122	72.131	27.869	0	photo	0.699
sll1373	8	100.000	0.000	0	photo	0.534
sll1396	12	83.333	16.667	0	photo	0.711
sll1401	8	87.500	12.500	0	photo	0.923
sll1476	1	100.000	0.000	0	photo	0.654
sll1531	26	96.154	3.846	0	photo	0.664
sll1665	22	81.818	18.182	0	photo	0.671
sll1714	26	100.000	0.000	0	photo	0.654
sll1717	23	73.913	26.087	0	photo	0.661
sll1730	28	89.286	10.714	0	photo	0.671
sll1755	17	100.000	0.000	0	photo	0.654
sll1761	11	100.000	0.000	0	photo	0.654
sll1763	35	82.857	17.143	0	photo	0.745
sll1764	36	97.222	2.778	0	photo	0.798
sll1765	8	100.000	0.000	0	photo	0.906
sll1830	53	98.113	1.887	0	photo	0.817
sll1853	19	100.000	0.000	0	photo	0.666
sll1863	51	96.078	3.922	0	photo	0.659
sll1885	35	100.000	0.000	0	photo	0.654
sll5002	11	81.818	18.182	0	photo	0.692
sll5006	10	80.000	20.000	0	photo	0.697
sll5044	9	55.556	44.444	0	photo	0.732
sll5069	3	66.667	33.333	0	photo	0.638
sll5089	20	95.000	5.000	0	photo	0.660
sll5090	7	100.000	0.000	0	photo	0.654
sll5109	3	66.667	33.333	0	photo	0.638
sll6010	7	100.000	0.000	0	photo	0.654
sll6069	7	100.000	0.000	0	photo	0.654
sll7009	23	100.000	0.000	0	photo	0.654
sll7043	30	86.667	13.333	0	photo	0.769
sll7062	18	83.333	16.667	0	photo	0.674
sll7063	37	100.000	0.000	0	photo	0.654
sll7064	6	100.000	0.000	0	photo	0.654
sll7066	41	75.610	24.390	0	photo	0.712
sll7067	24	100.000	0.000	0	photo	0.654
sll7070	26	96.154	3.846	0	photo	0.860
sll7085	25	96.000	4.000	0	photo	0.663
sll7086	10	100.000	0.000	0	photo	0.654
sll7087	37	94.595	5.405	0	photo	0.658
sll7089	26	96.154	3.846	0	photo	0.660
sll7090	31	100.000	0.000	0	photo	0.654
sll8007	22	90.909	9.091	0	photo	0.681
sll8011	45	91.111	8.889	0	photo	0.716
sll8017	1	100.000	0.000	0	photo	0.654

sll8033	18	100.000	0.000	0	photo	0.654
slr0019	18	77.778	22.222	0	photo	0.700
slr0059	28	100.000	0.000	0	photo	0.717
slr0061	17	100.000	0.000	0	photo	0.654
slr0103	11	72.727	27.273	0	photo	0.673
**slr0145**	26	100.000	0.000	0	photo	0.884
slr0196	23	100.000	0.000	0	photo	0.828
slr0209	66	96.970	3.030	0	photo	0.655
slr0226	80	97.500	2.500	0	photo	0.945
slr0262	24	87.500	12.500	0	photo	0.617
slr0271	9	100.000	0.000	0	photo	0.654
slr0272	10	80.000	20.000	0	photo	0.662
slr0386	66	71.212	28.788	0	photo	0.650
slr0398	16	87.500	12.500	0	photo	0.649
slr0421	67	64.179	35.821	0	photo	0.758
slr0442	136	62.500	37.500	0	photo	0.821
slr0496	15	73.333	26.667	0	photo	0.715
slr0522	22	86.364	13.636	0	photo	0.676
slr0569	21	80.952	19.048	0	photo	0.681
slr0572	10	100.000	0.000	0	photo	0.654
slr0573	8	100.000	0.000	0	photo	0.654
slr0579	8	100.000	0.000	0	photo	0.654
slr0581	15	86.667	13.333	0	photo	0.676
slr0582	8	100.000	0.000	0	photo	0.654
slr0602	10	80.000	20.000	0	photo	0.924
slr0667	9	100.000	0.000	0	photo	0.654
slr0668	8	100.000	0.000	0	photo	0.654
slr0727	10	70.000	30.000	0	photo	0.693
slr0881	89	76.404	23.596	0	photo	0.638
slr0912	21	66.667	33.333	0	photo	0.729
slr0913	8	87.500	12.500	0	photo	0.689
slr0914	12	91.667	8.333	0	photo	0.668
slr0937	53	71.698	28.302	0	photo	0.658
slr1107	15	100.000	0.000	0	photo	0.663
slr1135	19	73.684	26.316	0	photo	0.725
slr1168	113	93.805	6.195	0	photo	0.801
slr1187	9	100.000	0.000	0	photo	0.785
slr1210	84	53.571	46.429	0	photo	0.767
slr1222	9	100.000	0.000	0	photo	0.654
slr1232	10	90.000	10.000	0	photo	0.681
slr1243	90	53.333	46.667	0	photo	0.656
slr1396	13	84.615	15.385	0	photo	0.640
slr1397	8	100.000	0.000	0	photo	0.654
slr1398	7	100.000	0.000	0	photo	0.654
slr1421	35	100.000	0.000	0	photo	0.685
slr1437	43	100.000	0.000	42	photo	0.654
slr1450	19	100.000	0.000	0	photo	0.654
slr1544	35	97.143	2.857	0	photo	0.658
slr1552	26	100.000	0.000	0	photo	0.654
slr1567	37	94.595	5.405	0	photo	0.893
slr1571	84	79.762	20.238	0	photo	0.811
slr1576	9	88.889	11.111	0	photo	0.807
slr1616	119	85.714	14.286	0	photo	0.608
slr1670	36	83.333	16.667	0	photo	0.660
slr1773	32	100.000	0.000	0	photo	0.654
slr1774	36	77.778	22.222	0	photo	0.672
slr1778	12	100.000	0.000	0	photo	0.548
slr1788	65	98.462	1.538	0	photo	0.657
slr1789	65	98.462	1.538	0	photo	0.657
slr1869	9	88.889	11.111	0	photo	0.673
slr1920	10	100.000	0.000	0	photo	0.663
slr1959	56	64.286	35.714	0	photo	0.717
slr2018	39	100.000	0.000	0	photo	0.823
slr2037	21	71.429	28.571	0	photo	0.658
slr5013	21	80.952	19.048	0	photo	0.634
slr5016	46	86.957	13.043	0	photo	0.677
slr5073	11	100.000	0.000	0	photo	0.641
slr5085	3	100.000	0.000	0	photo	0.654
slr5126	2	100.000	0.000	0	photo	0.654

slr6004	1	100.000	0.000	0	photo	0.654
slr6005	13	92.308	7.692	0	photo	0.677
slr6006	14	92.857	7.143	0	photo	0.676
slr6007	49	63.265	36.735	0	photo	0.780
slr6013	6	83.333	16.667	0	photo	0.702
slr6014	10	70.000	30.000	0	photo	0.729
slr6015	7	100.000	0.000	0	photo	0.654
slr6016	11	100.000	0.000	0	photo	0.654
slr6021	8	100.000	0.000	0	photo	0.654
slr6022	4	100.000	0.000	0	photo	0.654
slr6028	10	80.000	20.000	0	photo	0.676
slr6031	17	76.471	23.529	0	photo	0.706
slr6033	1	100.000	0.000	0	photo	0.654
slr6045	3	100.000	0.000	0	photo	0.711
slr6063	9	88.889	11.111	0	photo	0.685
slr6064	13	92.308	7.692	0	photo	0.677
slr6065	14	92.857	7.143	0	photo	0.676
slr6066	49	63.265	36.735	0	photo	0.780
slr6072	6	83.333	16.667	0	photo	0.702
slr6073	10	70.000	30.000	0	photo	0.729
slr6074	7	100.000	0.000	0	photo	0.654
slr6075	11	100.000	0.000	0	photo	0.654
slr6080	8	100.000	0.000	0	photo	0.654
slr6081	4	100.000	0.000	0	photo	0.654
slr6087	10	80.000	20.000	0	photo	0.676
slr6090	15	86.667	13.333	0	photo	0.684
slr6091	2	100.000	0.000	0	photo	0.654
slr7010	103	59.223	40.777	0	photo	0.727
slr7011	30	96.667	3.333	0	photo	0.659
slr7061	15	93.333	6.667	0	photo	0.676
slr7080	28	100.000	0.000	0	photo	0.654
slr7081	8	62.500	37.500	0	photo	0.667
slr7099	2	100.000	0.000	0	photo	0.751
slr7100	10	90.000	10.000	0	photo	0.688
ssl0467	31	87.097	12.903	0	photo	0.675
ssl0750	8	100.000	0.000	0	photo	0.654
ssl0787	76	88.158	11.842	0	photo	0.576
ssl1326	18	55.556	44.444	0	photo	0.742
ssl1533	26	92.308	7.692	0	photo	0.656
ssl2138	66	96.970	3.030	0	photo	0.758
ssl2245	34	97.059	2.941	0	photo	0.663
ssl2420	55	87.273	12.727	0	photo	0.692
ssl2501	25	100.000	0.000	0	photo	0.654
ssl2507	37	100.000	0.000	0	photo	0.654
ssl3142	10	100.000	0.000	0	photo	0.821
ssl3222	15	93.333	6.667	0	photo	0.772
ssl3383	15	86.667	13.333	0	photo	0.673
ssl3615	18	72.222	27.778	0	photo	0.645
ssl3769	25	100.000	0.000	0	photo	0.654
ssl5001	20	100.000	0.000	0	photo	0.513
ssl5007	1	100.000	0.000	0	photo	0.654
ssl5065	6	83.333	16.667	0	photo	0.683
ssl5091	1	100.000	0.000	0	photo	0.654
ssl5096	32	87.500	12.500	0	photo	0.691
ssl5098	11	90.909	9.091	0	photo	0.646
ssl5103	15	93.333	6.667	0	photo	0.666
ssl6023	17	88.235	11.765	0	photo	0.660
ssl6035	11	100.000	0.000	0	photo	0.654
ssl6082	17	88.235	11.765	0	photo	0.660
ssl6092	20	55.000	45.000	0	photo	0.732
ssr0693	11	90.909	9.091	0	photo	0.651
ssr1038	25	96.000	4.000	0	photo	0.666
ssr1049	36	69.444	30.556	0	photo	0.695
ssr1853	26	76.923	23.077	0	photo	0.692
ssr2049	255	65.098	34.902	0	photo	0.902
ssr2153	11	100.000	0.000	0	photo	0.654
ssr2194	32	100.000	0.000	0	photo	0.654
ssr2317	51	80.392	19.608	0	photo	0.668
ssr2406	23	100.000	0.000	0	photo	0.654

ssr2422	50	78.000	22.000	0	photo	0.696
ssr2975	12	100.000	0.000	0	photo	0.573
ssr3159	8	87.500	12.500	0	photo	0.667
ssr3465	8	100.000	0.000	0	photo	0.654
ssr3532	54	88.889	11.111	0	photo	0.653
ssr5074	3	100.000	0.000	0	photo	0.654
ssr6002	2	100.000	0.000	0	photo	0.654
ssr6003	10	70.000	30.000	0	photo	0.747
ssr6048	9	77.778	22.222	0	photo	0.693
ssr7035	1	100.000	0.000	0	photo	0.654
slr9203	5	60.000	40.000	0	photo	0.768
slr9102	5	80.000	20.000	0	photo	0.696
slr9002	1	100.000	0.000	0	photo	0.654

References

1. Nowicka, B. & Kruk, J. Powered by light: Phototrophy and photosynthesis in prokaryotes and its evolution. *Microbiol Res* **186-187**, 99-118, doi:10.1016/j.micres.2016.04.001 (2016).
2. Pradella, S. *et al.* Genome organization and localization of the pufLM genes of the photosynthesis reaction center in phylogenetically diverse marine Alphaproteobacteria. *Appl. Environ. Microbiol.* **70**, 3360-3369, doi:10.1128/AEM.70.6.3360-3369.2004 (2004).
3. Vesth, T., Lagesen, K., Acar, Ö. & Ussery, D. CMG-Biotools, a Free Workbench for Basic Comparative Microbial Genomics. *PLoS One* **8**, e60120, doi:10.1371/journal.pone.0060120 (2013).
4. Enright, A. J., Van Dongen, S. & Ouzounis, C. A. An efficient algorithm for large-scale detection of protein families. *Nucleic Acids Res.* **30**, 1575-1584 (2002).
5. Frech, C. & Chen, N. Genome-wide comparative gene family classification. *PLoS One* **5**, e13409, doi:10.1371/journal.pone.0013409 (2010).
6. Gu, Q., Zhu, L. & Cai, Z. in *ISICA 2009: Computational Intelligence and Intelligent Systems.* 461-471 (Springer Berlin Heidelberg).
7. Shi, L. *et al.* The MicroArray Quality Control (MAQC)-II study of common practices for the development and validation of microarray-based predictive models. *Nat. Biotechnol.* **28**, 827-838, doi:10.1038/nbt.1665 (2010).
8. Matthews, B. W. Comparison of the predicted and observed secondary structure of T4 phage lysozyme. *Biochim. Biophys. Acta - Protein Structure* **405**, 442-451, doi:https://doi.org/10.1016/0005-2795(75)90109-9 (1975).
9. Wegener, K. M. *et al.* High sensitivity proteomics assisted discovery of a novel operon involved in the assembly of photosystem II, a membrane protein complex. *J. Biol. Chem.* **283**, 27829-27837, doi:10.1074/jbc.M803918200 (2008).
10. Ho, M. Y., Gan, F., Shen, G. & Bryant, D. A. Far-red light photoacclimation (FaRLiP) in Synechococcus sp. PCC 7335. II.Characterization of phycobiliproteins produced during acclimation to far-red light. *Photosynth Res* **131**, 187-202, doi:10.1007/s11120-016-0303-5 (2017).
11. Bussell, A. N. & Kehoe, D. M. Control of a four-color sensing photoreceptor by a two-color sensing photoreceptor reveals complex light regulation in cyanobacteria. *Proc Natl Acad Sci U S A* **110**, 12834-12839, doi:10.1073/pnas.1303371110 (2013).
12. Wiltbank, L. B. & Kehoe, D. M. Two Cyanobacterial Photoreceptors Regulate Photosynthetic Light Harvesting by Sensing Teal, Green, Yellow, and Red Light. *MBio* **7**, e02130-02115, doi:10.1128/mBio.02130-15 (2016).
13. Sanfilippo, J. E. *et al.* Self-regulating genomic island encoding tandem regulators confers chromatic acclimation to marine Synechococcus. *Proc Natl Acad Sci U S A* **113**, 6077-6082, doi:10.1073/pnas.1600625113 (2016).
14. Olsen, M. T. *et al.* The molecular dimension of microbial species: 3. Comparative genomics of Synechococcus strains with different light responses and in situ diel transcription patterns of associated putative ecotypes in the Mushroom Spring microbial mat. *Front Microbiol* **6**, 604, doi:10.3389/fmicb.2015.00604 (2015).
15. Rast, A., Rengstl, B., Heinz, S., Klingl, A. & Nickelsen, J. The Role of Slr0151, a Tetratricopeptide Repeat Protein from Synechocystis sp. PCC 6803, during Photosystem II Assembly and Repair. *Front Plant Sci* **7**, 605, doi:10.3389/fpls.2016.00605 (2016).
16. Knoppova, J., Yu, J., Konik, P., Nixon, P. J. & Komenda, J. CyanoP is Involved in the Early Steps of Photosystem II Assembly in the Cyanobacterium Synechocystis sp. PCC 6803. *Plant Cell Physiol* **57**, 1921-1931, doi:10.1093/pcp/pcw115 (2016).
